# The Role of Host-Generated H_2_S in Microbial Pathogenesis: New Perspectives on Tuberculosis

**DOI:** 10.3389/fcimb.2020.586923

**Published:** 2020-11-10

**Authors:** Md. Aejazur Rahman, Joel N. Glasgow, Sajid Nadeem, Vineel P. Reddy, Ritesh R. Sevalkar, Jack R. Lancaster, Adrie J. C. Steyn

**Affiliations:** ^1^ Africa Health Research Institute, Durban, South Africa; ^2^ Department of Microbiology, University of Alabama at Birmingham, Birmingham, AL, United States; ^3^ Department of Pharmacology and Chemical Biology, Vascular Medicine Institute, University of Pittsburgh School of Medicine, Pittsburgh, PA, United States; ^4^ Centers for AIDS Research and Free Radical Biology, University of Alabama at Birmingham, Birmingham, AL, United States

**Keywords:** H_2_S, hydrogen sulfide, *Mycobacterium tuberculosis*, immunometabolism, inflammation, CSE, CBS, 3-MST

## Abstract

For centuries, hydrogen sulfide (H_2_S) was considered primarily as a poisonous gas and environmental hazard. However, with the discovery of prokaryotic and eukaryotic enzymes for H_2_S production, breakdown, and utilization, H_2_S has emerged as an important signaling molecule in a wide range of physiological and pathological processes. Hence, H_2_S is considered a gasotransmitter along with nitric oxide (•NO) and carbon monoxide (CO). Surprisingly, despite having overlapping functions with •NO and CO, the role of host H_2_S in microbial pathogenesis is understudied and represents a gap in our knowledge. Given the numerous reports that followed the discovery of •NO and CO and their respective roles in microbial pathogenesis, we anticipate a rapid increase in studies that further define the importance of H_2_S in microbial pathogenesis, which may lead to new virulence paradigms. Therefore, this review provides an overview of sulfide chemistry, enzymatic production of H_2_S, and the importance of H_2_S in metabolism and immunity in response to microbial pathogens. We then describe our current understanding of the role of host-derived H_2_S in tuberculosis (TB) disease, including its influences on host immunity and bioenergetics, and on *Mycobacterium tuberculosis* (*Mtb)* growth and survival. Finally, this review discusses the utility of H_2_S-donor compounds, inhibitors of H_2_S-producing enzymes, and their potential clinical significance.

## Introduction

Although hydrogen sulfide (H_2_S) was not discovered until 1777 by the Swedish-German chemist Carl Wilhelm Scheele (Mitchell and Davenport, 1924), the description of its biological effects dates to the early 1700s, when Italian physician Bernardino Ramazzini (1633–1714) published his collection of observations regarding workers, their work environments, and occupation-associated illnesses as *De Morbis Artificum Diatriba* [Treatise on Worker’s Diseases]. Ramazzini is now considered “the father of occupational medicine,” and his compendium contains a chapter entitled, “Diseases of Cleaners of Privies and Cesspits” in which he described a “sewer gas” that caused painful irritation and inflammation in the eyes of sewer workers. Although Ramazzini didn’t know about the chemical nature of the species responsible for this, he noted that this inflammation could lead to bacterial infections and blindness (Felton, 1997; Szabo, 2018).

Until the late 1960s, H_2_S (we refer to H_2_S, HS^−^, and S^2−^ collectively as H_2_S or sulfide, unless specified otherwise) was still regarded as a foul-smelling toxic and flammable gas (Beauchamp et al., 1984) until it was understood to be produced endogenously in mammals. Indeed, three enzymes are responsible for the majority of H_2_S production in mammals: cystathionine γ-lyase (CSE), cystathionine β-synthase (CBS), and 3-mercaptopyruvate sulfurtransferase (3-MST). Another source of endogenous H_2_S is acid labile pools, which function in the presence of endogenous reductants and gut microbiota (Flannigan et al., 2011; Kimura, 2011). Notably, H_2_S is membrane-permeable and diffuses through cells (Mathai et al., 2009); therefore, it can act as a signaling molecule and/or interact directly with intracellular biomolecules.

H_2_S has since been increasingly recognized as an important physiological signaling molecule, along with nitric oxide (•NO) and carbon monoxide (CO) (Wang, 2014). H_2_S is now understood to have pleiotropic effects on human physiology. Indeed, it is an important mediator in cardiovascular (Kanagy et al., 2017; Pan et al., 2017) and respiratory systems (Bazhanov et al., 2017a), the nervous system (Kimura, 2019), and in inflammation and immunity (Bhatia, 2015). Notably, studies on the role of host H_2_S in bacterial (Benedetti et al., 2017; Saini et al., 2020; Rahman et al., 2020) or viral diseases (Pal et al., 2018), have been sparse. Therefore, it is reasonable to conclude that the role of host H_2_S in microbial diseases remains understudied.

The objective of this article is to review the physiological role of host-derived H_2_S in regulating various disease outcomes with an emphasis on its role in tuberculosis (TB). We begin by providing a brief description of H_2_S biochemistry and the enzymes that produce H_2_S, followed by the utility of H_2_S donor compounds and inhibitors of H_2_S production, which may have therapeutic value for the treatment of TB or other diseases. Next, we highlight the physiological importance of H_2_S in regulating mammalian metabolism and immunity in response to bacterial and viral infection. We then focus on the role of H_2_S in modulating immunity and metabolism, and how it contributes to TB pathogenesis. We conclude with brief overview of the role of endogenously produced H_2_S in bacterial physiology. For additional information, we refer the reader to several excellent review articles on the chemistry of H_2_S (Szabo, 2007; Mustafa et al., 2009; Wang, 2014; Filipovic et al., 2018; Szabo, 2018) and role of endogenous H_2_S in bacterial physiology (Shatalin et al., 2011; Mironov et al., 2017; Szabo, 2018; Toliver-Kinsky et al., 2019; Walsh and Giedroc, 2020).

## Overview of the Biochemistry and Biophysics of Sulfide

### Biochemical Properties

In biological molecules, sulfur can exist in a range of formal oxidation states, from −2 to +6, with H_2_S in the most reduced (−2) state. The biological chemistry of sulfur covalent compounds can be considered in terms of sulfur’s electronegativity (Sanderson, 1988). This means that its reactivity generally involves products that, relative to reactants, have increased electron density distributed away from the sulfur atom. As a nucleophile, sulfide thus reacts with electrophiles. Depending on the sulfur species and electrophilic reactant, electron redistribution can either be complete, resulting in transfer of an electron(s) from the sulfur-containing molecule to the electrophilic molecule, or partial. In the latter case, within a molecule, partial electron density is rearranged away from the sulfur and toward the electrophile but complete electron transfer does not occur. This sharing of electron density by the sulfur-containing nucleophilic moiety with the electrophilic moiety is a critical determinant of the covalent bond(s) between them, including strength and likelihood of formation.

Historically, oxygen has been the most important atom that, when bound to a nucleophile, results in significant “attraction” of electron density. This is why a change resulting in decreased electron density is referred to as “oxidation,” even if the reaction does not involve oxygen *per se*. The chemical reactions of sulfide are extensive (Filipovic et al., 2018) and the focus here will be on studies of reactions under physiologically relevant conditions that may be involved in signal transmission.

The protonation equilibria in aqueous solution are of critical importance in understanding the biochemistry of sulfide. Although there are two protonation equilibria for sulfide species,

(1) $${\text{H}}_{2}\text{S}↔{\text{H}}^{+}+\text{H}{\text{S}}^{-}$$

(2) $$\text{H}{\text{S}}^{-}↔{\text{H}}^{+}+{\text{S}}^{2-}$$

the pK_a_ of the second reaction is far above physiologically relevant pH (pK_a2_ = 17 to 19 (Filipovic et al., 2018)) so the sulfide anion (S^2−^) exists only in trace amounts. Since pK_a1_ is close to 7 under physiological conditions, both H_2_S and HS^−^ (which have different chemical profiles) are present in appreciable amounts. In addition, the volatility of H_2_S is an important parameter experimentally, as described below.

The first reported potential chemical mechanism of protein-mediated signal transmission by sulfide was the modification of protein cysteine to form persulfide (RSSH) (Mustafa et al., 2009). Although the mechanism proposed was S-sulfhydration (a misnomer (Toohey, 2012)), this is highly unlikely since both thiol and sulfide are nucleophiles and a reaction to form persulfide requires an oxidant. A far more likely reaction is H_2_S with disulfide (RSSR’) or sulfenic acid (RSOH) (Cavallini et al., 1970; Francoleon et al., 2011; Cuevasanta et al., 2015):

(3) H2S+RSSR'↔RSSH+R'SH

(4) $${\text{H}}_{2}\text{S}+\text{RSOH}\to \text{RSSH}+{\text{H}}_{2}\text{O}$$

Note that reaction (3) is reversible, implying that H_2_S and persulfide are at least theoretically kinetically interchangeable (Fukuto et al., 2020). Sensitive techniques have revealed the abundant presence of both protein and low molecular weight (cysteine and glutathione) persulfides in cells (Ida et al., 2014; Park et al., 2015; Fu et al., 2019). Akaike and colleagues have recently demonstrated the presence of enzymatic machinery that is capable of both sulfide-independent formation of free cysteine persulfide and direct translational incorporation of cys-SSH into newly synthesized proteins (Akaike et al., 2017). These findings have raised speculation as to whether the direct effector of signaling is sulfide, or whether a product from persulfide, or other polysulfide (Ida et al., 2014; Fukuto et al., 2018), or persulfide derivatives (Doka et al., 2020) is the functional entity (Alvarez et al., 2017; Filipovic et al., 2018; Fukuto et al., 2018).

In its interactions with other small reactive molecules, with the possible exception of hypochlorite (Nagy and Winterbourn, 2010), sulfide is unlikely to be an effective antioxidant under physiologically relevant (low concentration) conditions, although small amounts of oxidized sulfur species from such redox reactions may effect a signaling function as described above (Li and Lancaster, 2013; Nagy et al., 2014). The possibility of crosstalk between sulfide and nitric oxide (•NO) as biological signals (Fukuto et al., 2012; Kevil et al., 2017; Ivanovic-Burmazovic and Filipovic, 2019; Marcolongo et al., 2019) arose upon recognition of a signaling function for sulfide (Hosoki et al., 1997). In addition to overlapping and interactive biological downstream targets of •NO and sulfide (*e.g.*, vasodilation (Kimura, 2015)), several nitrogen oxide species react directly with sulfide and/or oxidized sulfur species to produce a variety of small reactive molecules, including thionitrous acid (HSNO), perthionitrite (ONSS^−^), polysulfides (HS_n_^−^), nitroxyl (HNO), and dinitrososulfite (ON(NO)SO_3_^−^; SULFI/NO) which is a diazeniumdiolate •NO/nitroxyl donor. These species could result in multiple effects including cysteine per- and poly-sulfidation (*vide supra*); however, the likelihood of their formation under biological conditions (with low concentrations of sulfide and •NO or alternative more abundant reactants) is not clear (Kevil et al., 2017). ONSS^−^ in particular has a relatively long lifetime and exhibits potent *in vivo* hypotensive activity, suggesting potential biological relevance (Cortese-Krott et al., 2015; Bogdandi et al., 2020).

In biological systems transition metal ions are strong electrophiles and interact with nucleophilic sulfur compounds, including sulfide. The focus here is on sulfide reactions with hemoproteins, since this interaction appears to be most important for TB (see section *The Role of Host-Derived* *H_2_S* *in Microbial Infections*). Prior to the discovery of sulfide signaling activity, its biomedical relevance was dominated by its toxicity, which, as first described by Keilin in 1929 (Keilin, 1929), is primarily due to inhibition of mitochondrial electron transfer at cytochrome *c* oxidase (C*c*O) (Petersen, 1977; Olson, 2012a; Nicholls et al., 2013). The mechanism of this inhibition is complex, involving as many as three sulfide molecules, acting both as an electron donor and a ligand to oxidized states of C*c*O, including ferric heme a_3_ (Cooper and Brown, 2008). Sulfide also reduces cytochrome *c*, providing electrons for C*c*O, and the sulfur oxidation product(s) increases protein persulfidation, thereby possibly potentiating sulfide signaling (Vitvitsky et al., 2018). A similar formation of sulfur oxidation products from endogenously produced sulfide has been reported for intact red blood cells, mediated by hemoglobin (Vitvitsky et al., 2015). Under oxidizing conditions, interaction of sulfide with hemoproteins can result in damaging covalent modification of the heme to form sulfheme, although this occurs only at relatively high sulfide levels (Filipovic et al., 2018).

There are numerous complex factors that determine the nature of the interaction of sulfide with hemoproteins, including the oxidation state of the heme iron, solution protonation state of the sulfide species and resultant solvent (H_2_O) interactions, dynamic configurations of bound heme and binding to water, access of the sulfide ligand to the distal heme pocket, and interaction of the bound ligand with amino acid residues and water (Pietri et al., 2011; Capece et al., 2013; Boubeta et al., 2020; Fukuto et al., 2020). In terms of ferroheme (not bound to protein) in a non-polar solvent, the hydrosulfide anion (HS^−^) can bind, but not H_2_S (Boubeta et al., 2020). The only well-documented ferrous heme sulfide complex in protein is myeloperoxidase (MPO), although no information was provided regarding the protonation state of the bound sulfide ligand (Palinkas et al., 2015). This apparent “exception” may be due to stabilization of bound ligand by an arginine residue in the heme pocket (Boubeta et al., 2020). Sulfide also acts as a reductant for MPO Complexes I and II.

The ferriheme protein that is undoubtedly best characterized for sulfide binding is the hemoglobin I of the bivalve mollusk *Lucina pectinata*, which delivers environmental sulfide as a respiratory substrate to a bacterial chemoautotrophic symbiont which provides organic carbon to the host (Kraus and Wittenberg, 1990; Kraus et al., 1990; Boubeta et al., 2016). As is true for essentially all ferric hemoproteins (including methemoglobin) it is protonated H_2_S that initially binds to the ferriheme, with non-existent or very weak binding by hydrosulfide (HS^−^). Experimental and theoretical evidence (Boubeta et al., 2020) suggests that this discrimination results from the inability of the hydrosulfide anion to access the heme as a result of the protein structure. The rate-limiting step in binding is release of the heme-bound water molecule prior to ligand binding. In some cases, heme-bound H_2_S deprotonates, leaving hydrosulfide as the final bound species which is stabilized by interaction with distal amino acid residue(s). The proximal heme ligand exerts major influence over the stability of the hydrosulfide complex, as well as the propensity of the sulfide to reduce the heme, forming ferroheme and oxidized sulfur species. Reduction and binding are also accomplished by persulfide interactions with heme proteins (Fukuto et al., 2020).

### Biophysical Properties

In the pure state and at standard temperature and pressure (STP) H_2_S is a gas. Unless exposed to a gaseous interface, this property is irrelevant to its biological actions (Fukuto et al., 2012; Li and Lancaster, 2013); however, it must be taken into account under certain experimental conditions (DeLeon et al., 2012). In a sealed container with headspace, at equilibrium the relative distribution of hydrogen sulfide (H_2_S) follows Henry’s Law, meaning its concentration in aqueous solution with a pure 1 atm H_2_S headspace is 110 mM and will decrease proportionately with partial pressure (Filipovic et al., 2018). The volatility of H_2_S under several laboratory physiological experimental conditions has been reported, with half-life ranging from 0.5 to 5 min (DeLeon et al., 2012).

Sulfide exhibits high turnover rates physiologically, resulting from a balance of production (by enzymes of the transsulfuration pathway, see *H_2_S-Producing Enzymes and Pathways*) and consumption (primarily *via* mitochondrial quinone reductase) with a resultant *in vivo* concentration in the 4–55 nM range (Kabil and Banerjee, 2014; Benchoam et al., 2019). As a small dissolved non-electrolyte similar to •NO and CO, hydrogen sulfide (H_2_S) is a highly lipophilic molecule that is freely membrane permeable and does not require facilitated diffusion (Mathai et al., 2009).

## H_2_S-Producing Enzymes and Pathways

**Figure 1:**
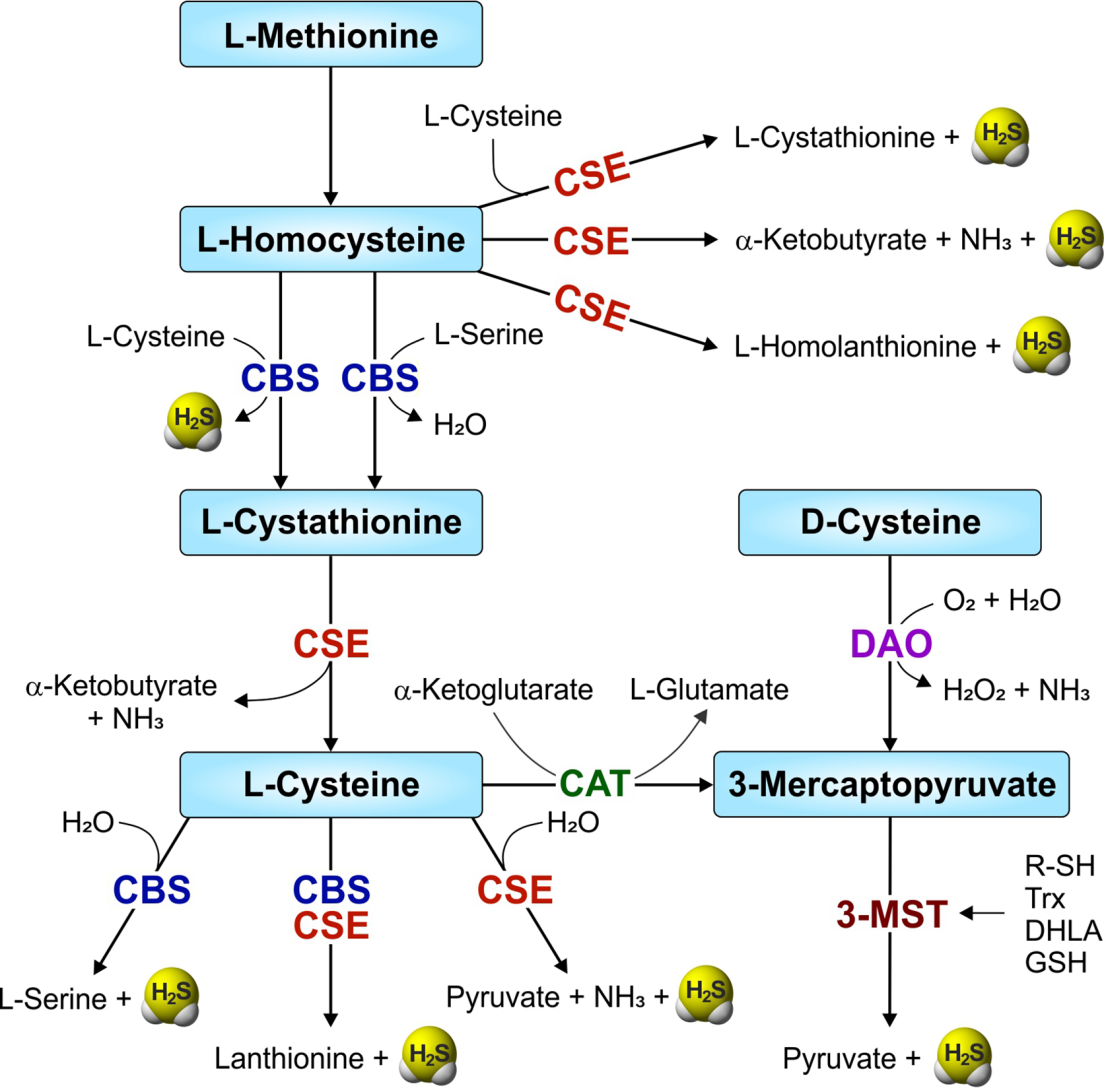
Overview of the mammalian transsulfuration pathway and H_2_S-producing enzymes. Two enzymes from the transsulfuration pathway—CBS (cystathionine-β-synthase) and CSE (cystathionine-γ-lyase), and another enzyme involved in sulfur metabolism, 3-MST (3-mercaptopyruvate sulfurtransferase) are involved in the production of H_2_S in mammals. CAT (cysteine aminotransferase) and DAO (D-amino acid oxidase) convert L-cysteine and D-cysteine respectively, to 3-mercaptopyruvate, a substrate for 3-MST. R-SH, thiols; Trx, thioredoxin (in the presence of NADPH/thioredoxin reductase).

The transsulfuration pathway involves the interconversion of cysteine and homocysteine through the intermediate cystathionine, leading to generation of sulfur metabolites and H_2_S. In mammals, H_2_S is synthesized within the transsulfuration pathway by two enzymes, namely cystathionine β-synthase (CBS; EC 4.2.1.22) and cystathionine γ-lyase (CSE; EC 4.4.1.1). Another enzyme involved in cysteine catabolism, 3-mercaptopyruvate sulfurtransferase (3-MST or MPST; EC 2.8.1.2) also produces H_2_S (**Figure 1**). CBS and CSE require the cofactor pyridoxal-5’-phosphate (PLP, the active form of vitamin B_6_) and catabolize L-cysteine to produce H_2_S within the transsulfuration pathway. However, H_2_S production by the PLP-independent enzyme 3-MST requires another PLP-dependent enzyme, cysteine aminotransferase (CAT, EC 2.6.1.3), to produce H_2_S from cysteine. Initially, CAT uses cysteine along with α-ketoglutarate to produce 3-mercaptopyruvate and L-glutamate. 3-MST then catalyzes the conversion of 3-mercaptopyruvate to pyruvate and H_2_S (Shibuya et al., 2009). However, the 3-MST reaction requires an additional reductant, such as thioredoxin (Trx) or dihydrolipoic acid (DHLA), for the release of H_2_S (Mikami et al., 2011). In contrast, a pathway which does not require PLP for H_2_S synthesis involves two enzymes, D-amino acid oxidase (DAO) and 3-MST. Here, DAO catalyzes D-cysteine to 3-mercaptopyruvate, the substrate for 3-MST (**Figure 1**) (Shibuya et al., 2013; Pollegioni et al., 2018).

### Cystathionine β-Synthase

The human CBS enzyme is a homotetramer comprised of ~63 kDa monomers. Each 551-amino acid monomer binds two cofactors, heme and PLP (the active form of vitamin B_6_), and is composed of three structural domains (Kery et al., 1994; Meier et al., 2001; Ereño-Orbea et al., 2013). The N-terminal domain consisting of residues 1–70 contains a heme-binding pocket, and the heme iron is axially coordinated by Cys^52^ and His^65^ (Meier et al., 2001; Taoka et al., 2002). While heme is not required for catalytic activity, it is required for protein folding, subunit assembly and for binding modulatory ligands CO and •NO. The ferric (Fe^3+^) state of heme iron is relatively inert and more stable, whereas the ferrous (Fe^2+^) state binds CO and •NO with different affinities (Puranik et al., 2006; Vicente et al., 2014). Both the oxidized (Fe^3+^) and reduced (Fe^2+^) state of the human CBS heme iron are low spin, hexa-coordinate species that are axially bound by the ligands His^65^ and Cys^52^. In the reduced heme state, binding of CO is hexa-coordinate by replacing the endogenous Cys^52^ ligand, whereas binding of •NO with heme results in a penta-coordinate species by replacing both ligands. Binding of CO or •NO with heme reduces CBS activity (Taoka and Banerjee, 2001; Vicente et al., 2014). Further, CBS activity is dependent on the oxidation state of the heme iron, as shown by a two-fold reduction in enzyme activity when the heme iron is in the reduced, ferrous (Fe^2+^) form, compared to the ferric form, which suggests the possibility of redox-associated regulation (Taoka et al., 1998; Taoka et al., 2002; Banerjee and Zou, 2005). The role of heme in regulating CBS catalytic activity is still unclear. However, a CBS deletion mutant lacking the 69 N-terminal residues does not bind heme, and retains only 40% activity (Evande et al., 2004).

The N-terminal domain is followed by a highly conserved catalytic domain that spans residues 71–413 and contains the PLP binding site (Banerjee and Zou, 2005). The C-terminal regulatory domain (residues 414–551) is comprised of tandem “CBS domains” (CBS1 and CBS2), a structural motif known for adenosine nucleotide binding and regulating protein activity *via* both intrasteric and allosteric effects (Bateman, 1997; Miles and Kraus, 2004; Baykov et al., 2011). CBS catalytic activity is modulated *via* binding of S-adenosyl-L-methionine (AdoMet or SAM) to two sets of binding sites in the regulatory domain (Finkelstein et al., 1975; Pey et al., 2013); AdoMet binding increases CBS activity 2–3 fold (Taoka et al., 1999) *via* stabilization of the protein (Prudova et al., 2006). The C-terminal regulatory domain is also critical for maintaining the tetrameric structure of CBS, as tryptic cleavage at Arg^413^ converts CBS to a dimer of core enzymes (Kery et al., 1998). Notably, C-terminal deletion mutants that lack the regulatory domain lose AdoMet responsiveness, but exhibit increased enzyme activity compared the full-length tetramer form, indicating that the C-terminal regulatory domain serves an autoinhibitory function (Kery et al., 1998; Evande et al., 2004; Banerjee and Zou, 2005).

CBS-mediated H_2_S production occurs *via* at least three reactions: 1) converting cysteine to serine and H_2_S, 2) condensing cysteine and homocysteine to yield cystathionine and H_2_S, and 3) condensing two cysteine molecules to lanthionine and H_2_S (Kabil et al., 2011; Giuffre and Vicente, 2018; Majtan et al., 2018). CBS can also catalyze cystine (the oxidized dimer form of cysteine) to form cysteine persulfide, a cysteine with its sulfhydryl group covalently bound to sulfur known as sulfane sulfur (Cys-SSH), pyruvate, and NH_3_ (Yadav et al., 2016). However, the first and committed step in the mammalian transsulfuration pathway, catalyzed by CBS, is the formation of L-cystathionine and water from the condensation of L-serine and L-homocysteine (Singh et al., 2009; Giuffre and Vicente, 2018; Majtan et al., 2018). Homocysteine is a toxic intermediate in the methionine cycle and the first molecule to enter the transsulfuration pathway for the formation of cysteine (Miles and Kraus, 2004; Singh et al., 2009). AdoMet, an allosteric activator of CBS, is another important intermediate in the methionine cycle that controls the metabolic flux between the transmethylation and transsulfuration routes (Finkelstein et al., 1975; Banerjee and Zou, 2005; Giuffre and Vicente, 2018). CBS is generally considered a cytoplasmic protein, but can be translocated to the nucleus (Kabil et al., 2006) and mitochondria (Bhattacharyya et al., 2013; Teng et al., 2013). While CBS is a major contributor to H_2_S synthesis throughout the central nervous system (Robert et al., 2003), CBS is also expressed in the liver, kidney, and pancreas (Bao et al., 1998; Kabil et al., 2011; Giuffre and Vicente, 2018).

### Cystathionine γ-Lyase

CSE is a homotetrameric enzyme composed of ~44 kDa monomers. Each 405-amino acid monomer consists of two structural domains. The larger N-terminal domain spans residues 9–263, contains the PLP binding pocket and is followed by the smaller C-terminal domain. The PLP cofactor is bound in the active site mainly by Lys^212^, and Tyr^60^ and Arg^62^ from the adjacent subunit (Sun et al., 2009). CSE is localized to the cytoplasm (Ogasawara et al., 1994) and expressed in the cardiovascular system, liver, kidney and lungs and pancreas (Hosoki et al., 1997; Zhao et al., 2001; Yang et al., 2004). Under increased calcium levels or hypoxia, CSE can translocate from the cytosol to the mitochondria of vascular smooth muscle cells, resulting in H_2_S production within mitochondria (Fu et al., 2012).

CSE is the second enzyme in the transsulfuration pathway and can utilize L-cystathionine to form L-cysteine, α-ketobutyrate and ammonia. In addition, CSE catalyzes other H_2_S-generating reactions: 1) condensation of L-cysteine and L-homocysteine to produce L-cystathionine and H_2_S, 2) utilization of two L-cysteine molecules to produce L-lanthionine and H_2_S, 3) breakdown of L-cysteine into pyruvate, H_2_S and ammonia, 4) condensation of two molecules of L-homocysteine to generate L-homolanthionine and H_2_S, and 5) degradation of L-homocysteine to generate α-ketobutyrate, H_2_S, and ammonia (Chiku et al., 2009; Singh et al., 2009; Giuffre and Vicente, 2018). In addition, CSE can catalyze cystine to form Cys-SSH, pyruvate and NH_3_, and homocystine (the oxidized dimer of homocysteine) to form homocysteine persulfide (Hcy-SSH), α-ketobutyrate, and NH_3_ (Yadav et al., 2016). Unlike CBS, CSE can generate H_2_S solely from homocysteine, an intermediate of the methionine cycle, as well as from cysteine alone. Under V_max_ conditions at saturating concentrations (10 mM cysteine or 30 mM homocysteine) CSE forms H_2_S at a 5-fold faster rate from homocysteine than from cysteine *via* condensation of two homocysteine molecules (reaction 4 above) (Chiku et al., 2009). However, under physiological conditions (10 μM homocysteine and 100 μM cysteine) over 70% of the H_2_S produced by CSE is predicted to come from cysteine (reaction 3 above), due to limiting homocysteine concentrations. However, at homocysteine concentrations of 40 μM and above that can occur in hyperhomocysteinemia, CSE is predicted to be the main contributor to H_2_S production where between 60 and 78% of H_2_S is derived from homocysteine alone (Chiku et al., 2009; Singh et al., 2009).

### 3-Mercaptopyruvate Sulfurtransferase

3-MST is the third enzyme in the cysteine catabolism pathway. It is thought to be evolutionarily related to the mitochondrial enzyme thiosulfate sulfurtransferase, known as rhodanese, as 3-MST contains two catalytic active rhodanese-like domains (RLD) (Nagahara et al., 1995). 3-MST is expressed in two isoforms. The full-length 317 amino acid isoform (3-MST-iso1) is comprised of a 20 amino acid N-terminal extension, followed by a 25-residue mitochondrial targeting sequence (MTS), RDL1 (residues 46–162), and RDL2 (residues 167–297) (Frasdorf et al., 2014). 3-MST-iso2 lacks the 20-residue N-terminal extension, exposing the MTS to localize this isoform to the mitochondria and cytoplasm, whereas 3-MST-iso1 is confined to the cytoplasm.

In the cysteine catabolism pathway, CAT converts L-cysteine and α-ketoglutarate into glutamate and 3-mercaptopyruvate (3-MP), as stated above. 3-MP is also generated from D-cysteine by D-amino acid oxidase (DAO) (Shibuya et al., 2013; Pollegioni et al., 2018). 3-MST then transfers the sulfur atom from 3-MP to a nucleophilic Cys^248^ (the catalytic site in human 3-MST) to generate a 3-MST-bound persulfide and pyruvate. This 3-MST bound persulfide (oxidized form) remains catalytically inactive until after release of H_2_S which is mediated by a reductant, such as reduced Trx, DHLA, glutathione (GSH), L-cysteine, L-homocysteine or by non-physiological reductants like 2-mercaptoethanol and dithiothreitol (DTT) (Nagahara and Katayama, 2005; Mikami et al., 2011; Yadav et al., 2013).

Unlike CBS and CSE, 3-MST is regulated primarily *via* its redox sensitivity and exists as a catalytically active monomer or an inactive disulfide-linked homodimer. This inter-subunit disulfide bond can be cleaved by a reducing agent such as reduced Trx, DHLA, GSH, or DTT, acting like a switch to activate 3-MST (Nagahara et al., 2007). Further, 3-MST can be inactivated *via* oxidation of solvent-exposed cysteines by hydrogen peroxide (H_2_O_2_); however, enzymatic activity can be restored in the presence of reducing agents DTT or reduced Trx (Nagahara and Katayama, 2005).

3-MST is involved in a broad range of physiological processes and can generate H_2_S and thiolate molecules in the cytosol and mitochondria. For example, 3-MST is responsible for detoxifying cyanide (CN^−^) by converting it to thiocyanate (SCN-), a less toxic molecule that can be safely metabolized and excreted (Nagahara et al., 1999). In the mouse brain, 3-MST has been shown to produce Cys-SSH, as well as glutathione (GSH) persulfide (GSSH), persulfurated cysteine residues on proteins, and H_2_S_2_. These sulfur-containing species play a dominant role in signaling and redox homeostasis (Kimura, 2015; Kimura et al., 2017). 3-MST is also referred to as transfer RNA (tRNA) thiouridine modification protein 1 (TUM1) due to its role in thiolation of cytosolic tRNAs. Thiolation of uridine at position 2 stabilizes the tRNA structure and ensures accurate mRNA decoding (Frasdorf et al., 2014). 3-MST is expressed in the perivascular glial cells in the brain, bronchiolar epithelial cells in the lung, myocardial cells in the heart, pericentral hepatic cells in the liver, and proximal renal tubular cells in the kidney (Nagahara et al., 1998).

## H_2_S Research Tools: Inhibitors of H_2_S-Synthesizing Enzymes and H_2_S Donor Compounds

### Inhibitors of H_2_S-Synthesizing Enzymes

As mentioned above, there are three H_2_S-producing enzymes in mammals: CBS, CSE, and 3-MST. The therapeutic potential of reducing endogenous H_2_S levels by inhibiting the activity of these enzymes has been evaluated in various diseases in small animal models and humans (Szabo, 2007; Vandiver and Snyder, 2012). The most commonly used inhibitors of H_2_S-producing enzymes are discussed below.

#### Aminooxyacetic Acid

Aminooxyacetic acid (AOAA) was the first pharmacological inhibitor of CBS to be widely used. AOAA is an inhibitor of CBS (IC_50_ of ~8.5 μM) and CSE (IC_50_ of ~1.1 μM) (Asimakopoulou et al., 2013). As described in detail below in section *Role of Host H_2_S in Tuberculosis*, data from *in vitro* and *in vivo* studies of *Mtb* infection demonstrate that AOAA reduces H_2_S production in host cells to reduce H_2_S-stimulated *Mtb* growth. Indeed, AOAA treatment of *Mtb*-infected peritoneal macrophages *in vitro* reduced *Mtb* growth compared to untreated controls. Further, intraperitoneal (IP) administration of AOAA to *Mtb*-infected WT mice reduced lung *Mtb* burden to the level observed in the lungs of *Cbs* ^+/−^ mice (Saini et al., 2020). Consequently, inhibiting H_2_S production warrants further evaluation as a potential therapeutic strategy for controlling TB.

AOAA has been employed extensively to study the role of H_2_S in cancer. While AOAA is not selective for CBS, it remains a useful tool for blockade of CBS-derived H_2_S *in vitro* and in animal models. The colon cancer-derived epithelial cell line HCT116 exhibits upregulation of CBS and increased H_2_S production compared to non-cancerous colon cells. Inhibition of CBS activity with AOAA reduced H_2_S production, reduced basal cellular respiration, suppressed ATP synthesis, reduced the spare respiratory capacity of HCT116 cells, and inhibited their growth. Further, administration of AOAA to mice bearing colon cancer xenografts slowed tumor growth (Szabo et al., 2013). AOAA treatment inhibited oxygen consumption, reduced ATP levels, and suppressed the proliferation of MDA-MB-231 breast adenocarcinoma cells compared to normal human mammary epithelial cells. In addition, AOAA treatment decreased the growth of pancreatic ductal adenocarcinoma cells (Son et al., 2013), ovarian cancer cell lines *in vitro* (Bhattacharyya et al., 2013), and MDA-MB-231 breast tumors in nude mice (Thornburg et al., 2008), emphasizing both the role of CBS-derived H_2_S in cancer pathophysiology and the therapeutic potential of inhibiting CBS enzymatic activity. While AOAA has shown promising results as an anti-cancer treatment in small animal models, it also exhibits dose-dependent toxicity in humans, indicating the need for further design and formulation for future studies.

#### DL-Propargylglycine

DL-Propargylglycine (PAG) is a selective, irreversible inhibitor of CSE with an IC_50_ of 40 μM (Sun et al., 2009; Asimakopoulou et al., 2013). In *Mtb*-infected murine macrophages, PAG treatment reduced H_2_S production and bacillary burden, indicating that host-derived H_2_S can modulate *Mtb* survival in macrophages. Further, PAG increased glycolysis and mitochondrial respiration in *Mtb*-infected macrophages (Rahman et al., 2020). These observations suggest that excess H_2_S impedes glycolysis and mitochondrial respiration in host cells during infection and shows H_2_S to be a key regulator of host central energy metabolism during tuberculosis. The use of PAG in *Mtb*-infected macrophages *in vitro* is discussed in more detail in the sections below: *H_2_S Promotes Mtb Growth by Suppressing Pro-Inflammatory Cytokines*, *Host H_2_S Suppresses Glycolysis and Oxygen Consumption in Macrophages*, and *H_2_S Stimulates Mtb Growth and Metabolism*.

In a rat model of hemorrhagic shock, PAG inhibited H_2_S production which led to increased plasma TNF-α, IL-6, and iNOS levels and accelerated recovery of normal blood pressure (Mok et al., 2004; Mok and Moore, 2008). In a mouse model of endotoxemia, administration of PAG reduced lipopolysaccharide (LPS)-induced elevation of •NO in plasma, and reduced myeloperoxidase, a marker of tissue damage, in the lungs and pancreas (Collin et al., 2005; Li et al., 2005) suggesting an anti-inflammatory role for H_2_S. The effect of H_2_S on the production of chemokines has been investigated. In a mouse model of cerulean-induced acute pancreatitis and associated lung injury, the pro-inflammatory effects of H_2_S were mediated by release of MCP-1, MIP-1α, MIP-2 chemokines, an effect that could be blocked by PAG (Tamizhselvi et al., 2008). Similarly, in the cecal ligation and puncture (CLP) sepsis model, PAG treatment reduced mRNA and protein levels of pro-inflammatory cytokines IL-1β, IL-6, TNF-α in mice, indicating that H_2_S potentiates systemic inflammation in sepsis (Zhang et al., 2007b). Drawbacks associated with PAG are its relatively low potency and poor cell permeability, requiring the use of high concentrations for CSE inhibition that can also inhibit aspartate and alanine aminotransferases (Szabo, 2007; Asimakopoulou et al., 2013).

#### *D*-Penicillamine

*D*-penicillamine is a penicillin derivative and the *D*-isomer of dimethylated cysteine that was originally used to treat rheumatoid arthritis, and is now used to treat Wilson Disease due to its ability to chelate accumulated copper, which is characteristic of this genetic disorder (Suarez-Almazor et al., 2000; Litwin et al., 2019). *D*-penicillamine inhibits both CBS and CSE, but is approximately 30 times more selective against CSE (IC_50_ of 270 μM) (Brancaleone et al., 2016). In a model of arterial inflammation, administration of *D*-penicillamine exacerbated the TNF-α inflammatory response by significantly increasing the number of adherent leukocytes as determined by intravital microscopy (Brancaleone et al., 2016). *D*-Penicillamine can have additional effects unrelated to CSE inhibition. For instance, treatment of murine RAW264.7 macrophage cells with *D*-penicillamine activates these cells *via* direct binding of *D*-penicillamine to cell-surface aldehydes, resulting in increased production of cytokines TNF-α, IL-6, and IL-23 (Li and Uetrecht, 2009). Likewise, *D*-penicillamine was shown to activate macrophages and T-cells, undergirding the ability of this compound to induce autoimmunity in Brown Norway rats (Masson et al., 2004). Overall, *D*-penicillamine inhibits CSE and activates immune cells, in addition to other functions. It is possible that this FDA-approved compound can be repurposed for therapeutic use in TB or other diseases where reduction of H_2_S levels is warranted.

#### β-Cyanoalanine

β-Cyanoalanine (BCA) is a neurotoxic agent that, unlike PAG, is a reversible inhibitor that acts by transiently modifying the CSE apoenzyme (Whiteman and Winyard, 2011). BCA is a slightly more potent inhibitor of CSE (IC_50_ of 14 µM) compared to PAG (IC_50_ of 40 µM) (Asimakopoulou et al., 2013). BCA can inhibit CBS, but only at concentrations exceeding 1 mM (Papapetropoulos et al., 2009; Asimakopoulou et al., 2013). BCA-mediated suppression of endogenous H_2_S synthesis resulted in enhanced neutrophil adhesion and infiltration, and edema formation in rats. This study suggests an important role for endogenous H_2_S as a modulator of key components of acute inflammatory responses which might influence the leukocyte-endothelial cell interface (Zanardo et al., 2006). However, BCA also inhibited enzymes such as aspartate β-decarboxylase and alanine aminotransferase, similar to PAG (Alston et al., 1980; Cornell et al., 1984). Undoubtedly, BCA is useful pharmacological tool for studying H_2_S biology, however further pharmacokinetic analyses are required before more widespread therapeutic use is feasible.

#### L-Aminoethoxyvinylglycine

L-aminoethoxyvinylglycine (AVG) is a specific inhibitor of CSE with an IC_50_ of 1.0 μM, although its use is not widespread, likely due to its unclear mechanism (Asimakopoulou et al., 2013). AVG is a natural toxin discovered in fermentation broth that inhibited the growth of *Streptomyces cellulosae*, and was reported to inhibit enzymes in the transsulfuration pathway (Clausen et al., 1997). Asimakopoulou *et al*. showed that the IC_50_ of AVG against CSE is 1 µM, making AVG the most potent inhibitor for this enzyme. Notably, AVG has extremely high selectivity for CSE and does not inhibit CBS activity even at concentrations up to 1 mM (Asimakopoulou et al., 2013). AVG inhibits human CSE *via* slow, tight, reversible binding to the PLP cofactor in the active by forming a Schiff base bond (Steegborn et al., 1999). Despite having high selectivity for CSE over CBS, AVG has been shown to inhibit other PLP-dependent enzymes, which should be considered when using this compound.

#### Other Inhibitors

A novel 3-MST inhibitor, HMPSNE, was identified in a drug screen and exhibits an IC_50_ of ~2–30 μM under various conditions (Hanaoka et al., 2017; Augsburger et al., 2020). HMPSNE suppressed H_2_S production in a concentration-dependent manner and reduced the proliferation and migration of CT26 murine colon cancer cells. Further, HMPSNE exerted a bell-shaped effect on oxygen consumption rate and extracellular acidification rate in CT26 cells. These observations suggests that 3-MST is the primary enzymatic source of H_2_S in CT26 cell line (Augsburger et al., 2020). Corvino *et al*. identified an oxothiazolidine derivative (referred to as compound a2) that efficiently inhibits CSE at concentrations 100-fold lower than PAG in aortic rings *ex vivo*. Further, compound a2 binding is reversible, offering a further advantage over PAG (Corvino et al., 2016). Use of a novel, highly selective inhibitor of human CBS, CH0004, elevated cellular homocysteine and suppressed H_2_S production in a dose-dependent manner in cell lines and in liver cancer xenografts. In addition, CH0004 triggered ferroptosis in HepG2 hepatic carcinoma cells and substantially reduced *in vivo* tumor growth in a xenograft mouse model (Wang et al., 2018).

Inhibitors of H_2_S-synthesizing enzymes have provided valuable insight into the varied roles of H_2_S in biological systems. However, like all inhibitors, these compounds can have off-target effects, such as inhibition of protein synthesis and transamination activity, and their use requires care and appropriate controls. Clearly, development of improved inhibitors would greatly benefit current H_2_S research and future clinical applications.

### H_2_S Donor Compounds

As H_2_S has emerged as a critical regulator of numerous physiological mechanisms in humans, it has also become clear that a variety of human pathologies are associated with aberrant H_2_S levels that contribute to disease (Vandiver and Snyder, 2012; Kondo et al., 2013; Bhushan et al., 2014; Polhemus et al., 2014). In biological systems, H_2_S is produced endogenously by host enzymes or it can be provided through the use of H_2_S-releasing donor compounds. In this regard, the development of H_2_S-releasing compounds as potential therapeutic agents has gained considerable interest (Powell et al., 2018). Not surprisingly, several classes of H_2_S donors have been developed to investigate the role of H_2_S in disease, and are described in comprehensive reviews (Song Z. J. et al., 2014; Li et al., 2018; Zheng et al., 2018; Levinn et al., 2020). Below, we discuss H_2_S-donor platforms known to modulate host immunometabolism, which is a key determinant in protection against various diseases including TB.

#### Inorganic H_2_S Donors

NaHS and sodium sulfide (Na_2_S) have been widely used donors of hydrosulfide (HS^−^, see *Overview of the Biochemistry and Biophysics of Sulfide*) the deprotonated biologically active form of H_2_S. In a mouse model of burn- and smoke-induced acute lung injury, subcutaneous administration of NaHS reduced the levels of pro-inflammatory cytokines IL-1 β, IL-6, and IL-8 and increased the anti-inflammatory cytokine IL-10 (Esechie et al., 2008). Similarly, NaHS reduced neutrophil adhesion, attenuated expression of inflammatory mediators such as *Tnf*, *Cox2*, and *Icam1*, and preserved mitochondrial function in various animal models (Fiorucci et al., 2005; Johansen et al., 2006; Elrod et al., 2007; Zhu et al., 2007; Sun et al., 2016). The use of NaHS is widespread in studying the role of H_2_S in the pathophysiology of various diseases, and is more fully discussed throughout section *The Physiological Importance of H_2_S*.

Nambi *et al*. demonstrated that addition of NaHS restores defective recycling of mycothiol observed in *Mtb* mutants deleted for components of the membrane-associated oxidoreductase complex (MRC). NaHS also reversed the sensitivity toward oxidative stress and survival of these *Mtb* mutants in both IFN-γ-activated bone marrow-derived macrophages and in mice, indicating that H_2_S can play a crucial role in maintaining redox homeostasis in *Mtb* (Nambi et al., 2015). Of note, release of HS^−^ from NaHS in aqueous solution is nearly instantaneous and can result in locally toxic concentrations for a short duration. This lack of sustained, controlled release has been addressed by the development of organic H_2_S donor compounds which have shown considerable therapeutic potential (Caliendo et al., 2010; Vandiver and Snyder, 2012; Guo et al., 2013).

#### Organic H_2_S Donors

Lawesson's reagent, a sulfurization reagent used in organic synthesis, releases H_2_S upon hydrolysis, which was shown to reduce TNF-α, IL-1β, reduce myeloperoxidase activity, increase levels of GSH, and protect rats through activation of ATP-sensitive potassium (K_ATP_) channels (Nicolau et al., 2013). Derived from Lawesson’s reagent, GYY4137 is a water soluble slow-releasing H_2_S donor that more accurately mimics physiological H_2_S production (Li et al., 2008). Addition of GYY4137 to human cancer cell lines was shown to alter energy metabolism by increasing glycolysis and glucose uptake and inhibiting the excretion of lactate in cancer cells, possibly by suppressing anion exchanger and sodium/proton exchanger activity. This combination of effects led to intracellular acidification and subsequent cell death (Lee et al., 2014). Similarly, GYY4137 released H_2_S over several days to cause cell cycle arrest and apoptosis in human cancer cell lines and in tumor xenografts in mice, while having no effect on normal lung fibroblasts. These data indicate that prolonged exposure to low levels of H_2_S can selectively kill cancer cells (Lee et al., 2011).

A recent study showed that exposure of H_2_S-deficient *Cbs^+/−^* macrophages and mice to GYY4137 increased H_2_S levels that stimulated *Mtb* growth similar to that observed in WT control cells and mice (Saini et al., 2020). In addition, GYY4137-sourced H_2_S reduced pro-inflammatory cytokines, altered bioenergetics and increased *Mtb* growth in *Cse* ^−/−^ macrophages comparable to wild type (Rahman et al., 2020). These studies suggest the utility of GYY4137 for pharmacologically modulating H_2_S levels in TB. Additional examples of the use of GYY4137 in disease models are presented throughout sections *The Physiological Importance of H_2_S* and *Role of Host H_2_S in Tuberculosis*.

#### Mitochondria-Targeted H_2_S Donors

H_2_S is known to exert profound effects on cellular bioenergetics and mitochondrial function (Szabo et al., 2014). Two novel mitochondria-targeted slow release H_2_S donors, anethole dithiolethione (AP39) (Le Trionnaire et al., 2014) and hydroxythiobenzamide (AP123) (Gero et al., 2016) were generated by coupling H_2_S-donating dithiolethione to a mitochondria-targeting moiety (triphenylphosphonium; TPP+). Hyperglycemia alters mitochondrial membrane potential by significantly increasing the activity of the tricarboxylic acid (TCA) cycle and increasing production of superoxide leading to mitochondrial dysfunction. AP39 provided targeted H_2_S release within the mitochondria, which reversed hyperglycemia-induced bioenergetic defects, increased cell viability, and minimized the loss of mitochondrial DNA integrity in microvascular endothelial cells undergoing oxidative stress (Gero et al., 2016). In addition, AP39 and AP123 treatment reduced mitochondrial oxidative free radicals (Gero et al., 2016). Similarly, RT01, a novel derivative of AP39, reversed hyperglycemia-induced mitochondrial hyperpolarization, oxidant production, and increased synthesis of ATP, leading to restoration of mitochondrial function in murine brain microvascular endothelial cells (Waters et al., 2017).

#### Reactive Oxygen Intermediate-Activated H_2_S Donors

This class of H_2_S donor limits the release of H_2_S to situations where reactive oxygen intermediates (ROI) are present and the anti-inflammatory activities of H_2_S would be particularly beneficial. In this regard, Zhao *et al*. tested a series of arylboronate-functionalized thiocarbamate (PeroxyTCM) compounds. Upon exposure to H_2_O_2_, and to a lesser extent to superoxide and peroxynitrite, PeroxyTCM compounds release carbonyl sulfide (COS) which is quickly converted to H_2_S by ubiquitous carbonic anhydrase (Zhao and Pluth, 2016; Zhao et al., 2017). In a similar approach, Hu and colleagues developed a ROI-triggered H_2_S donor, NAB, wherein COS release (and conversion to H_2_S by carbonic anhydrase) is accompanied by release of a strong fluorophore, allowing for real-time monitoring of H_2_S release (Hu et al., 2019).

#### Thiol-Activated H_2_S Donors

Thiol-activated H_2_S-releasing agents release H_2_S upon reacting with thiol-containing molecules such as glutathione and cysteine. Isothiocyanates were first reported as cysteine-activated H_2_S donors with relatively low release efficiency (Martelli et al., 2014; Martelli et al., 2020). Isothiocyanates have been shown to suppress NF-κB-mediated inflammation (Shehatou and Suddek, 2016), upregulate Nrf2 signaling to protect against oxidation (Huang et al., 2013) and at higher concentrations, induce apoptosis in cancer cell lines by altering mitochondrial function (Sehrawat et al., 2016). Thiol-Activated *gem*-dithiol-based H_2_S donors (TAGDD) release H_2_S in the presence of thiols like glutathione (GSH). TAGDD were able to significantly reduce production of pro-inflammatory cytokines IL-1α, IL-1β, IL-6, TNF-α, GM-CSF, and G-CSF leading to reduced lung inflammation in a mouse model of RSV infection (Bazhanov et al., 2018). Acyl perthiol donors (Zhao et al., 2013), dithioperoxyanhydride (Powell et al., 2018), *N*-(benzoylthio)benzamides (Zhao et al., 2011), and arylthioamides (Martelli et al., 2013) are H_2_S donors triggered in the presence of cysteine and/or GSH. A major advantage of the thiol-activated class of H_2_S donors is that free, naturally occurring thiols such as glutathione are relatively abundant in mammals, which provide a continuous source for H_2_S release. However, it may prove problematic in clinical settings because some patient groups have reduced levels of glutathione (Wang Y. et al., 2013).

Considerable effort has been focused on designing, testing, and understanding the chemical properties of several classes of H_2_S donors in a variety of biological systems. A primary consideration is the slow, prolonged release of H_2_S to better mimic physiological conditions. However, significant gaps in our knowledge remain that hinder clinical use of H_2_S donors. Continued innovation with a focus on bioavailability and targeted release of H_2_S will be critical in developing H_2_S-releasing therapeutic agents for clinical studies.

## The Physiological Importance of H_2_S

**Figure 2:**
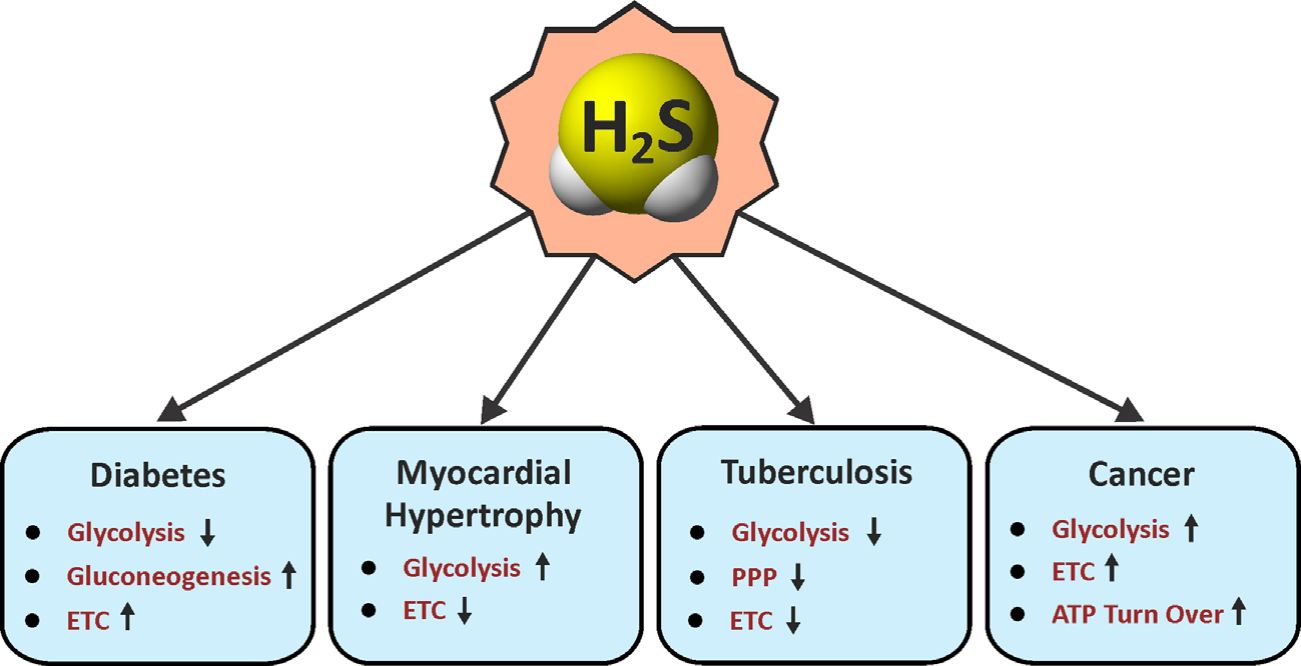
Roles of H_2_S in energy metabolism. Arrow indicates an H_2_S-mediated increase (up) or decrease (down) in the pathways associated with metabolism in various diseases. ETC, electron transport chain; PPP, pentose phosphate pathway.

**Figure 3:**
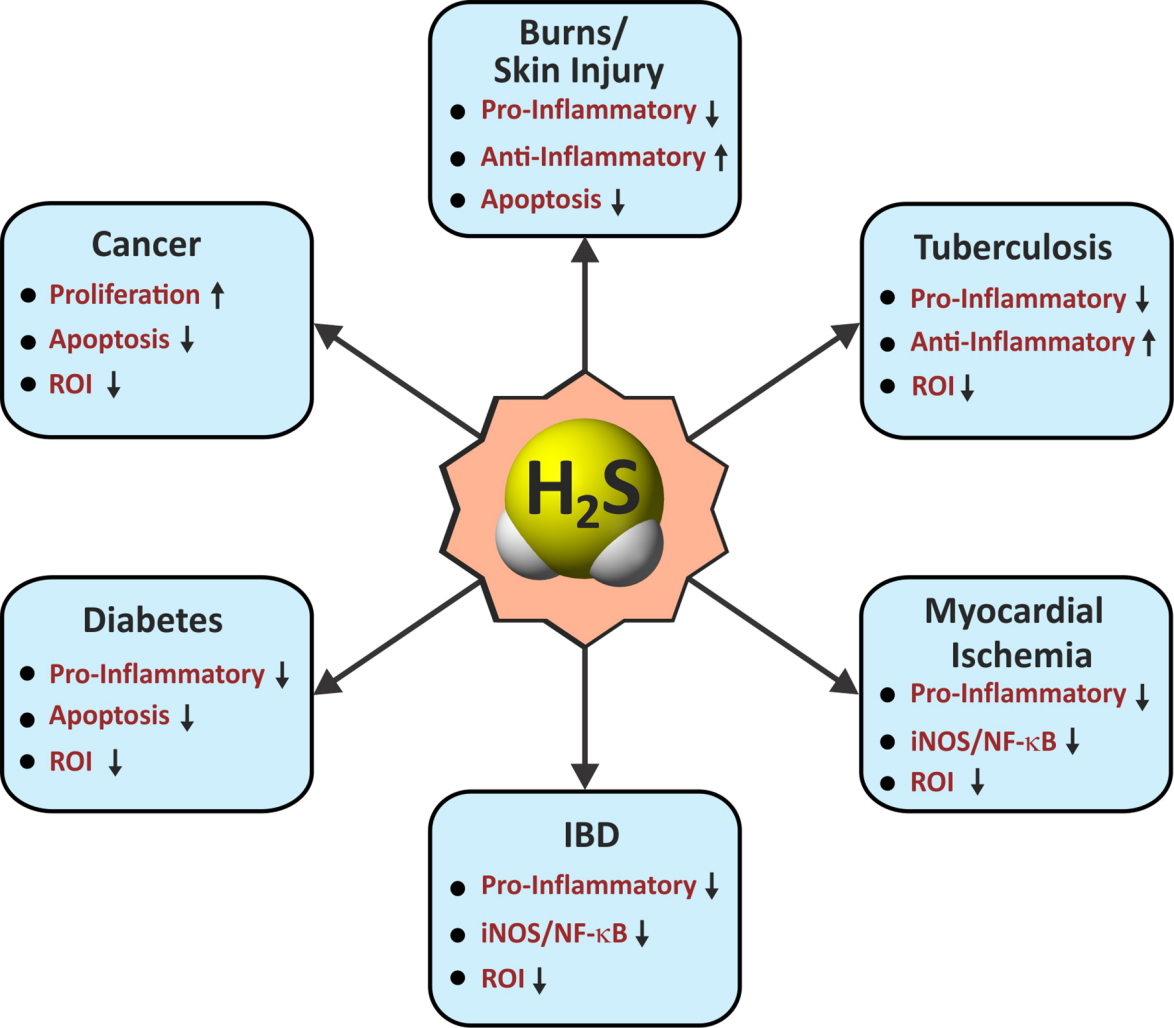
Roles of H_2_S in immunological pathways. Arrow indicates an H_2_S-mediated increase (up) or decrease (down) in the activity associated with immunological functions in various diseases. IBD, inflammatory bowel disease; Pro-Inflammatory/Anti-Inflammatory, cytokines; iNOS, inducible nitric oxide synthase; NF-κB, nuclear factor kappa B; ROI, reactive oxygen intermediates.

Since the discovery of endogenous production of H_2_S in mammals nearly three decades ago, H_2_S has gained considerable attention due to its physiological importance and therapeutic potential (Wang, 2012; Zhang Y. et al., 2013; Panthi et al., 2016). Although H_2_S was long known as a foul smelling and noxious gas, the biological role of H_2_S was never carefully examined until the end of the last century. Since then, similar to the gasotransmitters •NO and CO for which physiological roles were clearer, the role of H_2_S has been widely explored in numerous biological systems (**Figures 2** and **3**).

The first physiological role attributed to H_2_S was as a neuromodulator (Abe and Kimura, 1996) and neurotransmitter that regulates glutamate receptors, calcium ion concentrations, and cAMP levels (Kimura, 2000; Lu et al., 2008; Kamat et al., 2015). More recently, H_2_S has emerged as a therapeutic molecule in various central nervous system disorders such as Alzheimer’s disease, ischemia, and injury-related trauma (Zhang and Bian, 2014). Moreover, H_2_S has been implicated in vasodilation by inducing the relaxation of smooth muscle cells (Hosoki et al., 1997; Zhao et al., 2001; Teague et al., 2002; Zhao and Wang, 2002). Endogenous H_2_S has also been shown to play a role in regulating erectile dysfunction through relaxing the human corpus cavernosum (d’Emmanuele di Villa Bianca et al., 2009), and has been considered a potential anti-aging molecule by inhibiting free radical formation (Zhang Y. et al., 2013). Overall, a plethora of research has demonstrated a role for H_2_S in numerous pathophysiological processes such as diabetes (Wu et al., 2009; Szabo, 2012; Ma et al., 2017), hypertension (Wang et al., 2014; Sun et al., 2014; Meng et al., 2015), atherosclerosis (Wang et al., 2009; Zhang et al., 2012; Mani et al., 2014; Wang Z. J. et al., 2017), sepsis (Coletta and Szabo, 2013; Kosir and Podbregar, 2017; Qiu et al., 2018), and respiratory diseases such as asthma (Wang et al., 2011; Chung, 2014). H_2_S exerts its physiological functions in part through interacting with K+ ion channels (Jiang et al., 2010) and various signaling proteins. These effects are dependent on H_2_S concentration, which can vary from tissue to tissue or even from cell to cell (Olson, 2012b). Considering the diverse physiological roles of H_2_S, the following section is limited to a description of how H_2_S regulates various metabolic and immunological pathways in mammals.

### The Role of H_2_S in Metabolic Regulation and Disorders

Recent evidence suggests that metabolic programming of immune cells is tightly linked to immune cell function and fate (Al-Khami et al., 2017; Patel et al., 2019). Consequently, identifying the molecules, cytokines or microbial products that regulate these metabolic pathways to ultimately affect disease outcomes is the focus of active investigation. One of the first roles identified for H_2_S was the inhibition of cytochrome *c* oxidase (Complex IV), a component of the mitochondrial electron transport chain (ETC), which results in reduced ATP production under normoxic conditions (Blackstone et al., 2005). However, at low concentrations H_2_S increases oxygen consumption, membrane potential and mitochondrial ATP production (Lagoutte et al., 2010). H_2_S also serves as an oxygen sensor and energy substrate to regulate ATP production under hypoxic conditions (Fu et al., 2012). This function is mediated by a sulfide-quinone reductase whereby electrons derived from H_2_S are fed into the ETC to stimulate oxidative phosphorylation (OXPHOS) and mitochondrial ATP production (Hildebrandt and Grieshaber, 2008). Moreover, H_2_S helps maintain mitochondrial integrity by attenuating ROI and reactive nitrogen intermediates (RNI) (Suzuki et al., 2011).

An important mechanism by which H_2_S functions is through persulfidation of cysteines in target proteins, which can alter protein function (see *Overview of the Biochemistry and Biophysics of Sulfide*). Unlike nitrosylation, persulfidation accounts for 25–50% of the post-translational modifications of hepatic proteins (Jaffrey and Snyder, 2001; Mustafa et al., 2009). In 2009, Mustafa, *et al*. reported that 39 proteins in mouse liver lysates were persulfidated following treatment with sodium hydrosulfide (NaHS, a rapid releaser of H_2_S), several of which are involved in metabolic regulation. One vital enzyme regulated by persulfidation is glyceraldehyde-3-phosphate dehydrogenase (GAPDH), which is an important regulatory enzyme in glycolysis besides its role in gene transcription. This study showed that persulfidation of GAPDH at Cys^150^, which is critical for catalysis, enhanced its glycolytic activity by ~700%. Furthermore, a 25–30% reduction in GAPDH activity was observed in *Cse* ^−/−^ mice compared to WT controls, suggesting that endogenous H_2_S can modulate GAPDH activity (Mustafa et al., 2009). Interestingly, the α subunit (ATP5A1) of ATP synthase (Complex V) of the ETC is persulfidated by H_2_S at two positions, which significantly increases its enzymatic activity *in vitro* and *in vivo* (Modis et al., 2016).

Diabetes is a metabolic disorder resulting from impaired insulin secretion and/or insulin resistance that modulates carbohydrate metabolism (Kaneko et al., 2006; Al-Goblan et al., 2014). Several groups have demonstrated that obese participants or those with type 2 diabetes have significantly reduced plasma H_2_S levels, which is also seen in rodent models of type 1 diabetes (Jain et al., 2010; Whiteman et al., 2010a). However, in streptozotocin (STZ)-diabetic rats, increased *Cbs* and *Cse* mRNA and H_2_S formation were observed in the liver and pancreas (Yusuf et al., 2005), which regulates insulin secretion and resistance. Notably, increased pancreatic CSE expression and H_2_S production in the Zucker diabetic fatty (ZDF) rat model of diabetes was shown to reduce circulating insulin levels, resulting in hyperglycemia that could be reversed by administration of PAG (see *DL-Propargylglycine*) (Wu et al., 2009). Inhibition of insulin secretion by H_2_S is attributed to its ability to activate ATP-sensitive K+ channels (Yang et al., 2005). In another study, H_2_S was shown to impede insulin secretion by inhibiting Ca^2+^ channels (Kaneko et al., 2006). Tang *et al*. further substantiated that exogenous and endogenous H_2_S inhibits L-type voltage-dependent Ca^2+^ channels in pancreatic beta cells, and thus regulates insulin secretion in a mouse model (Tang et al., 2013). H_2_S may inhibit glucose metabolism by reducing intracellular accumulation of ATP, glucose transport, and mitochondrial oxidation in pancreatic beta cells (Kaneko et al., 2006) pointing to several mechanisms whereby H_2_S can influence insulin secretion in pancreatic beta cells.

H_2_S can also regulate liver metabolism, which may result in hepatic insulin resistance (Pichette and Gagnon, 2016). In a high fat diet-induced mice model of diabetes, reduced CSE expression led to lower H_2_S levels and decreased pyruvate carboxylase levels that inhibited gluconeogenesis and prompted glycolysis in the liver (Manna et al., 2014; Peh et al., 2014; Ju et al., 2015). In another study, H_2_S was reported to impair glucose uptake and increase gluconeogenesis in hepatocytes through increased activity of phosphoenolpyruvate carboxykinase and decreasing glucokinase activity (Zhang L. et al., 2013). The pathogenesis of diabetic complications is also associated with endothelial dysfunction which is linked to enhanced mitochondrial reactive oxygen species (ROI) (Giacco and Brownlee, 2010). H_2_S replacement therapy blocks the development of endothelial dysfunction by restoring oxidative phosphorylation in addition to improving mitochondrial depolarization, cellular ATP levels and reduced mitochondrial ROI production in hyperglycemic endothelial cells (Suzuki et al., 2011).

H_2_S has also been implicated in the regulation of cellular bioenergetics in various cancers. In primary ovarian carcinoma cells, a lack of H_2_S decreases mitochondrial oxygen consumption and enhances ROI production (Bhattacharyya et al., 2013). In colon cancer-derived epithelial cell lines, CBS levels are upregulated with concomitantly increased H_2_S production. Pharmacological inhibition of CBS in these colon cancer cell lines reduced cell proliferation, invasion, and migration along with suppressed glycolysis and mitochondrial function, suggesting that H_2_S can facilitate tumor growth (Szabo et al., 2013). In another study, increased levels of H_2_S in colonocytes activated the sulfide oxidation pathway and inhibited the ETC resulting in reductive stress as indicated by a reduced NAD^+^/NADH redox ratio (Libiad et al., 2019).

In summary, H_2_S regulates various facets of metabolism under different pathological conditions (**Figure 2**). Since metabolic processes are upstream of immune pathways that modulate immunological cell responses, H_2_S can have multiple roles in dictating disease outcomes.

### The Role of H_2_S in Immune Regulation

The immune system employs various effector mechanisms to protect against microbes and toxic substances. Not surprisingly, perturbations in immune regulation machinery are known to cause human disease. In this regard, numerous studies have shed light on the effects of exogenous or endogenous H_2_S in regulating immune responses (Wallace and Wang, 2015; Wallace et al., 2015; Fagone et al., 2018).

Considerable attention has been devoted to elucidating the role of H_2_S as a biological mediator of inflammation. Intriguingly, H_2_S has been shown to have a dual role in inflammatory processes, and both pro- and anti-inflammatory effects have been reported. The determining factor for a dual role of H_2_S in inflammation is unclear, but likely depends on the rate of H_2_S generation and/or H_2_S concentration (Whiteman et al., 2010b). Increased CSE activity and corresponding plasma H_2_S levels have been observed in hemorrhagic shock, pancreatitis, edema, and sepsis models of inflammation in mice (Mok et al., 2004; Bhatia et al., 2005; Li et al., 2005). In the cecal ligation and puncture-induced sepsis (CLP) model, administration of the CSE inhibitor PAG reduced leukocyte infiltration into tissues and increased the survival rate in mice. Also, decreased IL-6, TNF-α, and IL-1β levels were observed in the lungs and livers of these mice. Further, NaHS administration caused severe inflammatory damage through increased nuclear factor kappa B (NF-κB) activation (Zhang et al., 2007b; Zhang et al., 2007c). Importantly, LPS injection in mice has been shown to increase the plasma concentration of H_2_S through increased CSE activity. Inhibition of CSE through PAG administration in these mice reduced LPS-induced myeloperoxidase (MPO) activity in lungs and liver, which was accompanied by less leukocyte infiltration and tissue damage. Further, NaHS treatment led to a significant increase in plasma TNF-α levels, severe tissue damage in lungs, and increased MPO activity in both lungs and liver (Li et al., 2005). Administration of NaHS to the U937 macrophage cell line was shown to activate the p65 subunit of NF-κB and increase mRNA expression and protein levels of NF-κB target genes TNF-α, IL-6, and IL-1β (Zhi et al., 2007). Conversely, H_2_S can also exhibit anti-inflammatory functions by downregulating pro-inflammatory factors. For example, the H_2_S donor GYY4137 (Li et al., 2008), a slow releaser of H_2_S, and NaHS were shown to downregulate expression of the pro-inflammatory mediators TNF-α, ROI, and •NO in LPS-treated neuroblastoma cells and macrophage cells, illustrating the anti-inflammatory and cytoprotective role of H_2_S in LPS-mediated inflammation (Whiteman et al., 2010b; Yurinskaya et al., 2020). Also, the H_2_S-releasing compound ATB-429 reduced colitis-induced inflammation in mice by reducing granulocyte infiltration into colon tissue (Fiorucci et al., 2007). H_2_S also reduced gastric ulcer-related inflammation and promoted healing in a rat model (Wallace et al., 2007a). H_2_S-treated THP-1 cells incubated with lipid associated membrane proteins (LAMPs) from *Mycoplasma pneumonia* exhibited reduced production of pro-inflammatory cytokines IL-6 and IL-8 with a concomitant increase in HO-1 expression, suggesting an anti-inflammatory role for H_2_S (Hu et al., 2020).

Mice subjected to burn injuries had lower H_2_S plasma levels and increased levels of pro-inflammatory cytokines such as IL-6, IL-8 and TNF-α compared to control mice. Chemical complementation using NaHS reduced these cytokine levels and increased the anti-inflammatory cytokine IL-10 (Zeng et al., 2013). The H_2_S donor GYY4137 was shown to play a protective role against endotoxin-induced acute lung injury by decreasing iNOS activity and •NO release, and reduced leukocyte infiltration in lung tissues (Zhang et al., 2016). Further, GYY4137 has also been shown to alleviate diabetes-induced atherosclerosis by reducing oxidative stress, decreasing pro-inflammatory cytokines IL-1β, TNF-α, and IL-6, and suppressing the activation of the NLRP3 inflammasome (Zheng et al., 2019). In a rat model of myocardial ischemia-reperfusion injury, intermediate doses of NaHS (1.6 mg/kg) reduced expression of iNOS and NF-κB, and lowered oxidative stress and inflammation in heart tissue (Jeddi et al., 2020).

H_2_S has also been shown to be protective in various skin-related disorders such as psoriasis and Werner syndrome, an autosomal recessive disorder of premature aging. To model Werner syndrome, Werner fibroblasts were cultured, and it was noted that H_2_S-generating enzyme levels were lower, with increased oxidative stress and cytosolic aggregates, compared to normal cells. These phenotypic changes were reversed with NaHS and mTOR inhibitor treatment (Talaei et al., 2013). Psoriasis patients were found to have lower levels of serum H_2_S and higher TNF-α, IL-6, and IL-8 serum levels compared to healthy subjects. Treatment of HaCaT keratinocytes with exogenous H_2_S inhibited TNF-α-induced upregulation of IL-6, IL-8, and •NO, suggesting that H_2_S levels negatively correlate with disease severity (Alshorafa et al., 2012). Exogenous H_2_S supplementation reduced psoriasis symptoms and signs in a skin model of psoriasis (Rodrigues et al., 2015).

H_2_S has a cytoprotective function upon tissue injury in diabetic cardiomyopathy (DCM). NaHS administration in a rat model of DCM conferred protection from myocardial fibrosis through down-regulation of the JAK-STAT pathway, thereby suppressing inflammation, oxidative stress, and apoptosis (Liu M. et al., 2018). Moreover, in type 2 diabetes patients and in STZ-diabetic rats, lower levels of circulating H_2_S were reported compared to healthy counterparts, which was associated with increased vascular inflammation (Jain et al., 2010). To model the role of H_2_S in diabetes-related vascular complications, HUVEC cells were exposed to high glucose (25 mM) and underwent apoptosis associated with an increased Bax/Bcl-2 ratio, caspase-3 activation, and increased ROI, all of which was prevented by pre-treatment with NaHS (Guan et al., 2012). H_2_S also downregulates miRNA-194 and plays a protective role against fibrotic changes through collagen realignment in diabetic kidneys (John et al., 2017). H_2_S also improved outcomes in a mouse model of diabetes-associated cognitive decline (DACD), a diabetic complication resulting in cognitive impairment. In one study, administration of NaHS to diabetic mice improved spatial learning, which was associated with modulation of the mitochondrial apoptotic pathway as evidenced by reduced levels of Bax and reduced cleavage of caspase-3 and -9. Further, levels of IL17/IL-23 were decreased in these mice suggesting an overall anti-apoptotic and anti-inflammatory role for H_2_S (Ma et al., 2017).

Over the past few decades, there has been an upsurge in the prevalence of inflammatory bowel disease (IBD) and other immunologically linked human disorders owing to changes in diet and lifestyle. H_2_S has been implicated in IBD, however its role remains controversial. H_2_S has been reported to suppress the expression of pro-inflammatory cytokines including IFN-γ and TNF-α (Li et al., 2007; Wallace et al., 2007b). In a mouse model of dextran sodium sulfate (DSS)-induced colitis, CBS and CSE expression increased in the gut following DSS administration, and inhibition of the CSE with PAG worsened markers of inflammatory disease, suggesting that H_2_S has anti-inflammatory effects in colitis (Hirata et al., 2011).

H_2_S has been reported to modulate the expression of genes involved in apoptosis, cell cycle control, and proliferation in a concentration and cell type-dependent manner (Faller et al., 2010; Zhang J. H. et al., 2010; Baskar and Bian, 2011). In human lung fibroblasts, NaHS causes DNA damage and cell cycle arrest (G1) in a concentration-dependent manner. This was coupled with increased expression of Bax, p21, and cytochrome c and stabilization of p53 (Baskar et al., 2007). Similar observations were seen when H_2_S-treated human and rat aorta smooth muscle cells exhibited increased apoptosis (Yang et al., 2006; Baskar et al., 2008). Another study reported that H_2_S induced free radical–mediated genomic DNA damage in Chinese hamster ovary cells (Attene-Ramos et al., 2007). Hoffman *et al.* reported that micromolar concentrations of H_2_S caused genomic single-strand breaks as a result of ROI generated by the auto-oxidation of H_2_S (Hoffman et al., 2012). Thus, H_2_S plays a role in cell growth and proliferation and may be relevant in the development of cancer therapeutics.

In conclusion, it is clear that H_2_S represents a key signaling molecule that can modulate various processes such as inflammation, oxidative stress, apoptosis, proliferation, and more (**Figure 3**). Exploiting H_2_S to regulate these events is likely to alter disease outcome. Moreover, promising results from preclinical studies in diverse pathophysiological conditions including, but not limited to, diabetes, neurodegeneration and ophthalmic disorders, psoriasis, and sepsis highlight the therapeutic potential of H_2_S.

### Role of H_2_S in Macrophage Polarization

**Figure 4:**
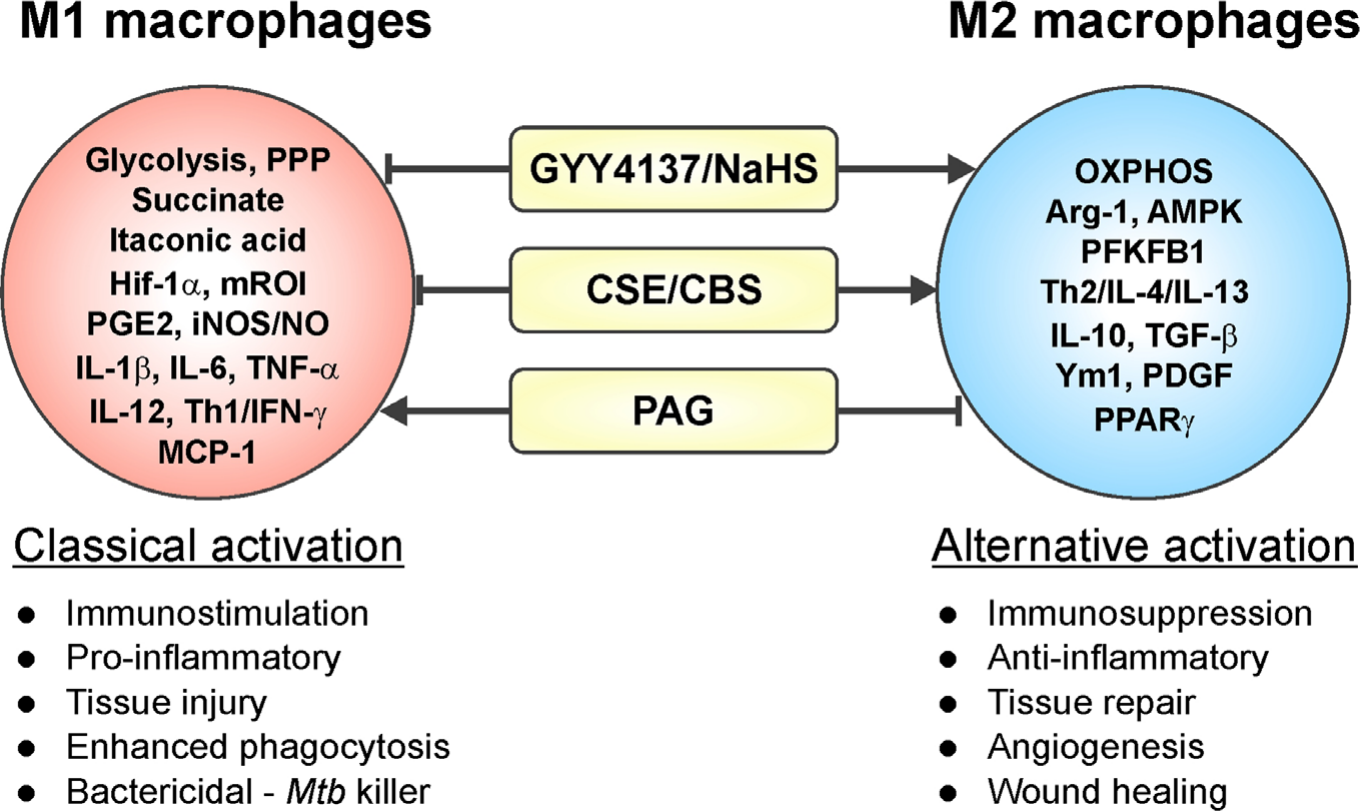
Involvement of H_2_S in macrophage polarization. Increased levels of H_2_S in an inflammatory macrophage model (caused by stimuli such as IFN-γ, LPS, or *Mtb* infection) trigger a phenotypic shift in macrophages leading to an increased M2 phenotype. On the other hand, reduced H_2_S levels induce a pro-inflammatory response, which drives macrophages toward an M1 phenotype. Hence, H_2_S can trigger M1 to M2 macrophage polarization with few exceptions.

Macrophages are critical effector cells of the innate immune response and can switch between two opposing immunological phenotypes: M1 (pro-inflammatory or classically activated) and M2 (anti-inflammatory or alternatively activated) (Sica and Mantovani, 2012; Sica et al., 2015; Viola et al., 2019). M1 polarization occurs by sensing microbial products (LPS) or stimulation with Th1 cytokines like IFN-γ or GM-CSF. M1 macrophages are characterized by an increased capacity for antigen presentation, a Hif-1α-mediated metabolic shift toward glycolysis and the pentose phosphate pathway (PPP) that ultimately yields ROI for killing pathogens, and secretion of pro-inflammatory mediators like IL-1β, IL-6, TNF-α, IL-12, RNI, and prostaglandin E_2_ (PGE_2_) (Sica and Mantovani, 2012; Sica et al., 2015; Khan et al., 2019; Viola et al., 2019). In contrast, M2 polarization can be induced by Th2 cytokines IL-4, IL-13, IL-10, TGF-β, and M-CSF. M2 macrophages are characterized by anti-inflammatory processes, including production of Arg-1, IL-10, TGF-β, IGF-1, and collagen to dampen inflammation and stimulate cell survival and tissue repair (Mantovani et al., 2013; Khan et al., 2019; Viola et al., 2019). M2 macrophages are more reliant on OXPHOS and have reduced rates of glycolysis (Mills and O’Neill, 2016). Numerous studies have reported that H_2_S modulates inflammatory processes, exerting both pro-inflammatory and anti-inflammatory effects (**Figure 4**). Specifically, H_2_S has been implicated in influencing macrophage phenotype, as discussed below.

A study carried out in the RAW264.7 cell line showed that GYY4137 inhibited the LPS-induced release of pro-inflammatory mediators IL-1β, IL-6, TNF-α, •NO, and PGE_2_, and increased synthesis of the anti-inflammatory cytokine IL-10 in a dose-dependent fashion (Whiteman et al., 2010b). These effects suggest that slow H_2_S release *via* GYY4137 can drive LPS-induced M1 macrophages toward an M2 phenotype. In the same study, addition of NaHS, which rapidly releases a bolus of H_2_S, showed a biphasic effect, with modest reductions in PGE_2_ at lower NaHS concentrations and increased IL-1β and TNF-α levels higher concentrations (200–1,000 µM) of NaHS (Whiteman et al., 2010b).

In a rat model of renal fibrosis based on unilateral ureteral obstruction, obstructed kidneys exhibited increased CSE expression, loss of CBS expression, and reduced H_2_S levels compared to controls. Obstructed kidneys also showed considerable macrophage infiltration and fibrosis. Notably, administration of NaHS (5.6 or 56 µg/kg/day, IP) attenuated inflammation by reducing macrophage infiltration and expression of inflammatory cytokines IL-1β, TNF-α, and MCP-1. In contrast, higher doses of NaHS (560 µg/kg/day, IP) increased inflammatory cytokine expression (Song K. et al., 2014).

Oxidized LDL (ox-LDL) is a major risk factor for developing atherosclerosis. Stimulation of macrophages *in vitro* with ox-LDL reduced CSE expression and H_2_S levels, and increased production of TNF-α. Conversely, overexpression of CSE or addition of NaHS reduced TNF-α production and endothelial cell adhesion in ox-LDL treated macrophages, showing that suppression of the CSE/H_2_S axis may be required to initiate and maintain a pro-inflammatory phenotype (Wang X. H. et al., 2013).

Further, exposure of microglia (macrophages in the central nervous system) to rotenone, a common pesticide and inhibitor of mitochondrial complex 1, leads to M1 polarization along with increased production of TNF-α, iNOS, and PGE_2_. M1 polarization coincided with reduced expression of CBS and lower H_2_S levels. However, overexpression of CBS in rotenone-treated microglia or exposure to NaHS (50, 100, 500 µM) caused reversion toward the M2 phenotype (Du et al., 2014).

Macrophages are critical for tissue repair following myocardial infarction (MI). In a mouse model of MI, macrophages from *Cse* ^−/−^ mice displayed an M1 phenotype with increased expression of IL-1β, IL-6 and TNF-α compared to WT controls. NaHS treatment of WT and *Cse* ^−/−^ mice reduced pathological cardiac remodeling with a reduction of infarct size. This was associated with an increased number of M2 macrophages that expressed IL-10, Arg-1, and Ym1 (Miao et al., 2016).

The studies mentioned above and numerous others have employed inhibitors of CSE and CBS (PAG and AOAA, respectively), L-cysteine and the H_2_S donors NaHS and GYY4137, as well as *Cse* ^−/−^ mice within various macrophage inflammatory models, which clearly established H_2_S as an effector molecule involved in resolution of inflammation by driving macrophages toward an anti-inflammatory M2 phenotype (Castelblanco et al., 2018; Zhou et al., 2019; Sunzini et al., 2020). Similarly, macrophages infected with *Mtb* exhibited increased CSE expression and H_2_S levels leading to an anti-inflammatory or M2 macrophage phenotype. This was reflected in increased *Mtb* growth compared to *Cse* ^−/−^ mice macrophages as discussed further in section *H_2_S and M2 Macrophage Polarization in Tuberculosis* (**Figure 4**). In contrast, a recent study of macrophage polarization during mechanical load-promoted tooth movement reported that load-stimulated periodontal ligament stem cells produce H_2_S that promotes M1 macrophage polarization and production of pro-inflammatory cytokines (He et al., 2020). Overall, it appears that upregulation of H_2_S-producing enzymes and/or exogenous H_2_S supplementation in inflammatory macrophages drives polarization toward an M2 phenotype with few exceptions.

## The Role of Host-Derived H_2_S in Microbial Infections

A large body of literature is available on the chemical biology of H_2_S and the role of H_2_S in diverse biological systems. However, studies on the role of H_2_S in microbial infection are limited. This section focuses on the contribution of H_2_S to the host response against bacterial and viral infections.

### Host-Derived H_2_S in Response to Bacterial Infections

Apart from studies aimed at elucidating the role of host-derived H_2_S in *Mtb* infection (discussed below in *Role of Host H_2_S in Tuberculosis*), little is known about the role of host-generated H_2_S in modulating the course of bacterial infections. However, studies using mouse models of septic shock have examined the role of H_2_S in response to lipopolysaccharide (LPS), an inflammatory cell wall component of Gram-negative bacteria. Notably, IP injection of LPS in mice increased CSE expression in liver and kidney resulting in increased levels of H_2_S in tissues and serum (Li et al., 2005). Lung sections of these LPS-treated animals exhibited characteristic signs of inflammatory damage, including interstitial edema, alveolar thickening, and the presence of numerous leukocytes in both the interstitium and alveoli. Inhibition of CSE enzyme activity with PAG triggered an anti-inflammatory effect in LPS-injected animals, thus providing indirect evidence that H_2_S exerts a pro-inflammatory effect in this model (Li et al., 2005). Another study performed in human macrophages shows that inhibition of NF-κB and ERK prevented LPS-induced increases in H_2_S, suggesting that H_2_S acts as an inflammatory mediator *via* the NF-κB/ERK pathway in macrophages (Badiei et al., 2015). A study by Ahmad *et al*., using mice deficient in H_2_S production (*Cbs* ^+/−^, *Cse* ^−/−^, and Δ*3-Mst* mice that exhibit decreased 3-MST expression in the lung and spleen) showed differential cytokine responses compared to WT following LPS injection. Plasma levels of multiple cytokines including TNFα, IL-1β, IL-2, IL-4, IL-5, IL-6, IL-10, IL-12, and IFN-γ were increased in WT mice upon LPS treatment whereas all three H_2_S-deficient mice showed decreased plasma levels of TNF-α, IL-10, IL-12, and IFN-γ. On the other hand, plasma levels of IL-5 and GM-CSF were increased in H_2_S-deficient mice while the levels of IL-1β, IL-2, IL-4, and IL-6 were similar to WT mice (Ahmad et al., 2016). However, survival of all three H_2_S-deficient mice following LPS administration was the same as WT mice.

In the case of *Mycoplasma fermentans* infection of macrophages, it was shown that H_2_S inhibits the activation and nuclear translocation of NF-κB, reducing the transcription of pro-inflammatory genes, including MCP-1 (Benedetti et al., 2014). Moreover, *M. fermentans* infection enhances Nrf2 functions by activating downstream enzymes including HO-1 and SOD1, and by decreasing intracellular ROI levels (Benedetti et al., 2017). H_2_S also inhibits Keap1 by persulfidation at Cys^151^, which allows nuclear translocation of Nrf2 and transcriptional activation of cytoprotective genes *via* binding to an antioxidant/electrophile response element (ARE) in target gene promoters (Hourihan et al., 2013). Persulfidation of Keap1 is important in protecting the host against oxidative stress and cellular senescence, which are generally observed in viral or bacterial infection (Hourihan et al., 2013). Overall, the literature suggests that H_2_S acts by modulating cytokine responses and by modifying host transcription factors to promote bacterial clearance.

### Host H_2_S in Response to Viral Infections

Several studies reported that H_2_S has important antiviral and anti-inflammatory activity in respiratory syncytial virus (RSV) infection, since the virus reduced the expression levels of H_2_S-producing enzymes (Li et al., 2015; Ivanciuc et al., 2016). Intranasal delivery of GYY4137 to RSV-infected mice significantly reduced viral replication and markedly improved clinical disease parameters and pulmonary dysfunction. Similarly, *Cse* ^−/−^ mice showed significantly worse RSV-induced lung disease and increased viral replication compared to WT mice (Ivanciuc et al., 2016). RSV infection of A549 cells leads to activation of two transcription factors, NF-κB and IRF-3. Addition of exogenous H_2_S to RSV-infected cells leads to a decrease in the levels of these transcription factors, suggesting a role for H_2_S in regulating NF-κB and IRF-3 (Li et al., 2015). Exogenous H_2_S using GYY4137 significantly reduced RSV replication *in vitro* as well as *in vivo* by targeting viral assembly, replication, and virion release. PAG treatment led to an increase in RSV-induced cytokines IL-6, IL-8, IL-10, and chemokine MIP-1β, and also increased viral replication (Li et al., 2015). A similar effect of H_2_S was observed during infection with other members of the paramyxoviridae family; Nipah virus (NiV-B) and human metapneumovirus (hMPV) (Ivanciuc et al., 2016). The replication of other pathogenic enveloped RNA viruses from the Orthomyxo-, Filo-, Flavi-, and Bunyavirus families has also been shown to be affected by H_2_S (Bazhanov et al., 2017b). This same group showed that GYY4137 treatment led to decreased expression of viral proteins and mRNA, suggesting inhibition of an early step of replication. It was reported that the antiviral activity of H_2_S was due to a decrease of the virus-induced pro-inflammatory mediators NF-κB and IFN-γ (Bazhanov et al., 2017b).

Studies have established a link between H_2_S and Coxsackie virus B3 (CVB3) infection (Hua et al., 2009; Wu et al., 2015). CVB3 infects cardiomyocytes resulting in immune cell infiltration and cardiac remodeling, which can eventually result in heart failure (Wu et al., 2015). Administration of GYY4137 to CVB3-infected cardiomyocytes suppressed secretion of pro-inflammatory cytokines TNF-α, IL1β, and IL-6 (Wu et al., 2015). Similar to RSV infection, in the case of CVB3, exogenous H_2_S treatment inhibited NF-κB signaling by reducing degradation of IκBα. CVB3 infection leads to induction of MAPK signaling cascade by activating ERK1/2, p38, and JNK1/2. Exogenous administration of GYY4137 resulted in reduced induction of these pathways, suggesting an anti-inflammatory role for H_2_S in CVB3 infection (Wu et al., 2015).

The coronavirus disease 2019 (COVID-19) pandemic resulting from severe acute respiratory syndrome coronavirus 2 (SARS-CoV-2) infection is a global health emergency that has led to over 1 million deaths in over two hundred countries as of this writing (WHO, 2020). COVID-19 pathology is variable and patients exhibit a wide range of clinical symptoms (Esakandari et al., 2020; Zhou et al., 2020). COVID-19-related severe respiratory failure is characterized in part by increased numbers of neutrophils and lower numbers of lymphocytes (CD4, CD8, and CD19), as well as increased levels of serum IL-6 and C-reactive protein (CRP) (Giamarellos-Bourboulis et al., 2020; Zhou et al., 2020). It has been speculated that H_2_S may play a protective role against COVID-19-mediated pathology through several mechanisms: firstly, by altering the function or expression levels of angiotensin converting enzyme 2 (ACE2) and transmembrane protease serine 2 (TMPRSS2) receptors to impede SARS-CoV-2 entry into host cells. Secondly, by inhibiting viral replication through attenuation of virion assembly or release, and thirdly, by suppressing pulmonary immune responses and inflammation (Yang, 2020). A study of 74 COVID-19 patients reported that surviving patients had significantly higher H_2_S levels than non-survivors, and that serum H_2_S levels negatively correlated with serum IL-6, CRP, and procalcitonin (markers for bacterial infection, sepsis, and tissue injury) (Renieris et al., 2020). However, these findings should be interpreted with caution since the levels of H_2_S, which were measured by monobromobimane (MBB) followed by high-performance liquid chromatography, were unphysiologically high (Olson et al., 2014).

Altogether, the available information indicates that during viral infection, H_2_S modulates NF-κB signaling, which in turn can reduce the virus-induced pro-inflammatory response and improve host survival. Hence, these findings point toward H_2_S donors as potential therapeutics against viral infections.

### Host H_2_S and Sepsis

Severe sepsis and septic shock are a leading causes of mortality in intensive care units (Medam et al., 2017). Sepsis is caused by a severe systemic infection due to the presence of bacteria or their toxins in blood or tissue, which frequently occurs after hemorrhage, trauma, burn, or abdominal surgery. Studies using mouse models of sepsis [*e.g*., cecal ligation and puncture (CLP)] show increased expression of CSE in the liver and higher plasma H_2_S levels upon induction of sepsis (Zhang et al., 2006). Inhibition of H_2_S production with PAG significantly decreased sepsis-induced systemic inflammation. PAG pre-treatment also considerably reduced the phosphorylation of ERK1/2 in lung and liver 4 h after CLP, coupled with reduced degradation of IκBα and reduced NF-κB activation (Zhang et al., 2008).

H_2_S also contributes to neurogenic inflammation in the respiratory tract *via* induction of Substance P (SP) (Zhang et al., 2007a). SP is implicated in inducing the release of pro-inflammatory mediators, enhanced lymphocyte proliferation, and stimulating chemotaxis of lymphocytes, monocytes, and neutrophils *via* activation of the neurokinin-1 receptor (O’Connor et al., 2004). H_2_S was also shown to induce systemic inflammation and multiple organ damage characteristic of sepsis *via* transient-receptor potential-vanilloid-type-1-(TRPV1)-mediated neurogenic inflammation (Ang et al., 2010). The effect of TRPV1 was shown to be mediated through the enhancement of SP production and activation of the ERK/NF-κB pathway (Ang et al., 2011a).

Recent studies have shown that sepsis activates macrophages to produce PGE_2_, a potent inflammatory mediator, *via* induction of cyclooxygenase-2 (COX-2), which is important to evoke acute lung injury. H_2_S upregulates COX-2 and prostaglandin E metabolite (PGEM) in sepsis through the TRPV1 channel. Importantly, inhibition of COX-2 with parecoxib, a potent and selective COX-2 inhibitor, prevents H_2_S from exacerbating acute lung injury (ALI) and CLP-induced mortality in sepsis. Administration of H_2_S using NaHS further enhanced the biosynthesis of COX-2 and PGEM, whereas PAG significantly reduced these effects, which improved recovery of the septic injury (Ang et al., 2011b). Delivery of NaHS to sepsis-induced mice leads to a marked increase in lung pro-inflammatory cytokines TNF-α, IL-1β, IL-6, chemokines MIP-1α and MIP-2, and adhesion molecules P-selectin, E-selectin, VCAM-1, and ICAM-1 which affect inflammation and immune cell migration. The levels of cytokine and cell adhesion molecules were reduced upon treatment with parecoxib, which suggests that H_2_S can act *via* COX-2 (Ang et al., 2011b).

Other than affecting immune pathways, sepsis is also known to cause mitochondrial dysfunction (Brealey et al., 2002). In the pneumosepsis model, *i.e*., sepsis induced by *Streptococcus* *pneumonia*, it was reported that NaHS infusion reduced local and distant organ injury, which was associated with maintaining mitochondrial function and improved mitochondrial biogenesis (Aslami et al., 2013). In this study, rats were challenged with live *S. pneumonia* and infused with NaHS to study its effects on metabolism and bioenergetics. Infusion of NaHS reduced heart rate and body temperature, indicative of a hypo–metabolic state. NaHS infusion also reduced sepsis–related lung and kidney injury, increased expression of α–tubulin and protein kinase C-ϵ, which act as regulators of respiration (Rostovtseva et al., 2008; Nowak and Bakajsova-Takacsova, 2018), and help to prevent mitochondrial membrane damage during sepsis (Aslami et al., 2013).

In summary, the evidence presented here indicates that host-derived H_2_S often plays a pro-inflammatory role in animal models of sepsis, acting *via* SP, NK1R, or TRPV1. The activation of NK1R or TRPV1 induces systemic inflammatory response *via* the ERK-NF-κB pathway to induce an exaggerated immune response. In this regard, agents that attenuate H_2_S production and/or H_2_S-induced downstream signals improved outcomes. However, in cases where H_2_S is clearly anti-inflammatory and/or acts to prevent the loss of, or re-establish, host bioenergetic health by upregulating regulators of respiration like α–tubulin and protein kinase C-ϵ, the use of H_2_S donor compounds should be evaluated further.

## Role of Host H_2_S in Tuberculosis

*Mtb* is the etiological agent of pulmonary TB. In the lung, *Mtb* is phagocytosed by alveolar macrophages, where it subverts host killing mechanisms and innate immune responses that promote its survival and dissemination. Importantly, recent studies have established that the metabolic state of innate immune cells and the efficacy of their immune response are interrelated (Gleeson and Sheedy, 2016; O’Neill et al., 2016; Kumar et al., 2019; Van den Bossche et al., 2017). The gasotransmitters •NO and CO, in addition to more recently appreciated H_2_S, play a vital role in modulating central energy metabolism and effector functions of immune cells. Further, •NO and CO, produced by nitric oxide synthase II (iNOS) and heme oxygenase 1 (HO-1), respectively, are important host factors that can alter *Mtb* survival and TB disease progression (MacMicking et al., 1997a; MacMicking et al., 1997b; Kumar et al., 2007; Kumar et al., 2008; Chinta et al., 2016; Chinta et al., 2018). Despite functions that overlap with •NO and CO, and its involvement in pathophysiological processes, the role of host-generated H_2_S in bacterial pathogenesis has been largely overlooked. Inflammatory responses (Zumla et al., 2015; Liu Q.Y. et al., 2018) and metabolic reprogramming of innate and adaptive immune cells are associated with the progression of pulmonary TB (Shi et al., 2016; Kumar et al., 2019). Notably, similar pathophysiological roles are also associated with the effector functions of H_2_S, depending on concentration and cell type (Bhatia et al., 2005; Zhang et al., 2006; Zhang J. et al., 2010; Miller et al., 2012; Bhatia, 2015; Gaddam et al., 2016). Recent studies have provided insight into the role of host-derived H_2_S in affecting pathological and immunometabolic aspects of TB (Rahman et al., 2020; Saini et al., 2020). The role of the H_2_S-producing enzymes CBS and CSE in TB are described in more detail below.

### H_2_S-Producing Enzymes in Human Tuberculous Lungs

Immunohistochemical (IHC) analysis of lesioned and uninvolved human tuberculous lung tissue suggests that CBS is either absent or expressed at undetectable levels. However, variable expression of CSE and 3-MST was observed in TB lungs and depended on the cellular composition around the necrotic and non-necrotic granulomas (Rahman et al., 2020). In short, robust CSE staining was seen in myofibroblasts, epithelioid histiocytes (activated macrophages), giant cells, and other immune cells in the non-necrotic and the granulomatous inflammation layer of the necrotic granuloma. Also, vascular mural smooth muscles cells showed strong CSE staining. Alveolar pneumocytes and bronchiolar epithelial cells exhibited strong 3-MST staining. The bronchiolar epithelium was also weakly positive for CSE. In most CSE-positive cells, CSE was localized to cytoplasmic and nuclear compartments, but unlike CSE, 3-MST was seen mostly in the cytosol. Neither CSE nor 3-MST staining was noted in the adluminal necrotic components of the cavity wall or the central necrotic component of the granuloma. In healthy lung tissue, intense staining of CSE and weaker 3-MST staining was observed in alveolar pneumocytes, respiratory and terminal bronchiolar epithelium, circulating monocytes, scattered desquamated epithelial cells, and in vascular smooth muscle, while no CBS-positive cells were observed. Overall, the number of cells staining positive for CSE and 3-MST around TB lesions was markedly increased compared to healthy lung tissue, suggesting that increased H_2_S production is a host response to *Mtb* infection (Rahman et al., 2020).

### Role of CSE and CBS in Tuberculosis

**Figure 5:**
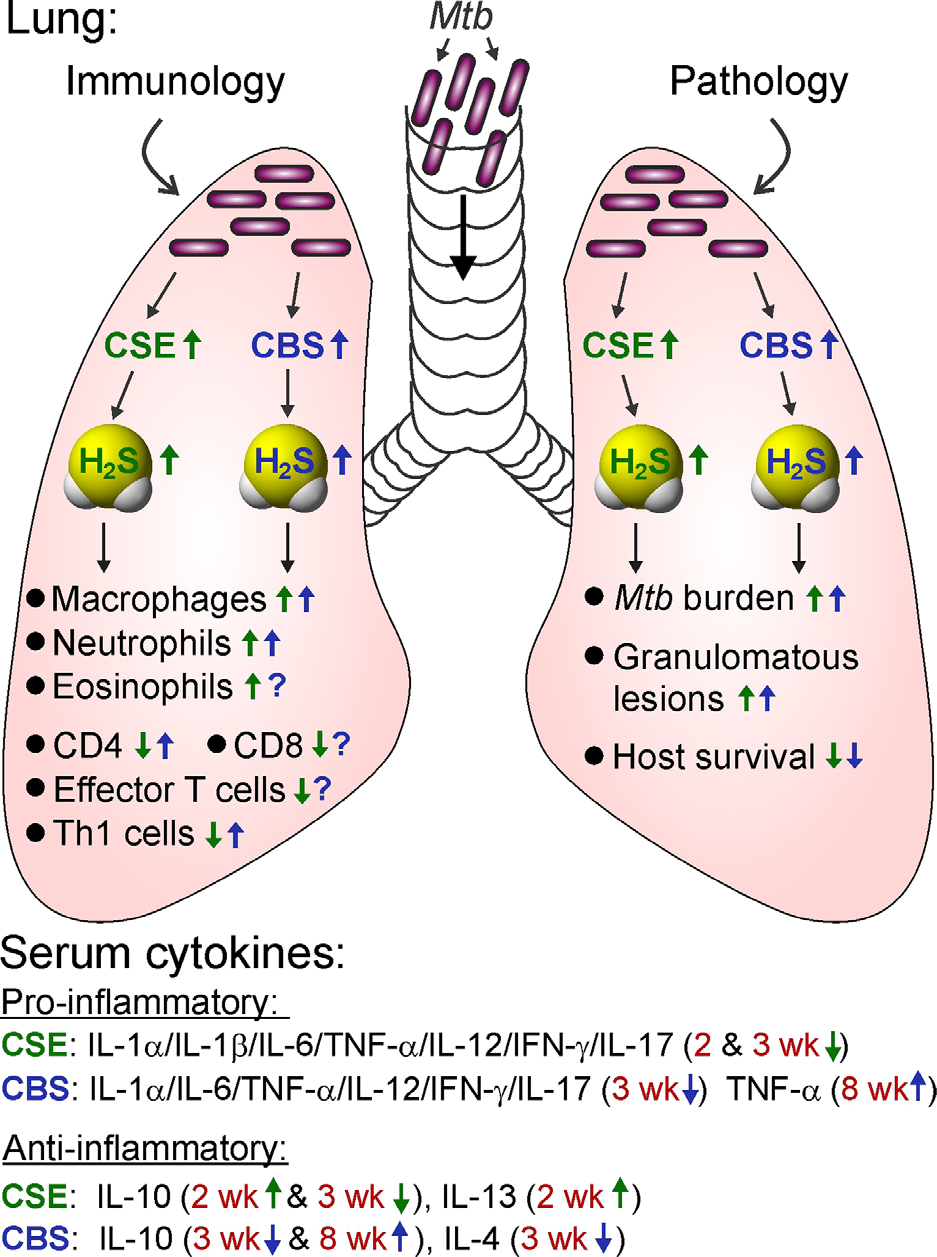
Role of CSE and CBS in the mouse model of TB. *Mtb* infection of the lung increases CSE and CBS levels, which increases the number of innate immune cells (macrophages, neutrophils, and eosinophils), regulates adaptive immune cells (CD4, CD8, effector T cells—IFNγ+Th1 cells) and serum cytokines. Increased CSE and CBS levels are associated with increased H_2_S production leading to increased *Mtb* burden, more granulomatous lesions, and reduced survival. Blue and green text and arrows indicate the regulation caused by CBS and CSE, respectively. Arrows indicate an H_2_S-mediated increase (up) or decrease (down) in the activity associated with TB disease. Question marks (?) indicate that the role of CBS is unknown. wk, observed time points in weeks.

Detailed characterization of TB pathology was performed in separate studies employing *Cse* knock-out (*Cse* ^−/−^) or *Cbs* heterozygous knockout (*Cbs* ^+/−^) mice. Since *Cbs* ^−/−^ mice exhibit early lethality, *Cbs* ^+/−^ mice were used which have a normal life span and ~50% reduction in CBS levels and H_2_S production (Watanabe et al., 1995; Saini et al., 2020). The course of TB disease was altered in both H_2_S-deficient mouse models, based on the following observations: i) *Mtb*-infected *Cse* ^−/−^ and *Cbs* ^+/−^ mice survived significantly longer than WT mice, ii) the *Mtb* organ burden in the lungs, spleens and livers of *Cse* ^−/−^ and *Cbs* ^+/−^ mice was significantly lower than in WT controls, iii) histopathological analysis of the lungs revealed that *Cse* ^−/−^ and *Cbs* ^+/−^ mice had less consolidated tissue than WT mice over time, and that these mice also had fewer and smaller granulomatous lesions than corresponding WT mice. Moreover, more bacilli were observed in the acid-fast stained lung sections in the WT mice than in the *Cse* ^−/−^, providing additional evidence for increased CFU in the WT mice. Lastly, iv) *Mtb* infection led to an increase in CBS and CSE protein levels in the lungs of *Cbs* ^+/−^ and *Cse* ^−/−^ mice as well as WT controls (Rahman et al., 2020; Saini et al., 2020) (**Figure 5**). Taken together, these data indicate that CSE and CBS exacerbate TB pathology.

### Immunological Characterization of *Cse* ^−/−^ and *Cbs* ^+/−^ Mice

As discussed above, CSE and CBS contribute to *Mtb* growth and disease progression. The difference in disease severity between WT and *Cse* ^−/−^ or *Cbs* ^+/−^ mice may result from altered immune responses. Immunological characterization showed no significant differences in the percentages of myeloid or lymphoid cell types in the lungs of *Cbs* ^+/−^ and WT mice prior to, or 3 weeks after infection with *Mtb*. However, 8 weeks after infection, WT mice exhibited increased percentages of neutrophils, macrophages, CD4^+^ cells, and CD4^+^IFN-γ^+^ cells in the lung compared to *Cbs* ^+/−^ mice (**Figure 5**). These data suggested that the increase in bacillary burden and decreased survival in WT mice may not result from immunological differences, but rather from the effects of host-derived H_2_S on *Mtb* (Saini et al., 2020). Unlike CBS, CSE was shown to contribute to immune dysregulation by promoting an excessive innate immune response and suppressing adaptive immune responses to *Mtb* infection in the lung (Rahman et al., 2020). The contributions of CSE and CBS to immune regulation in the context of *Mtb* infection are discussed in more detail below.

#### H_2_S and Regulation of Innate Immunity

Significantly increased numbers of alveolar macrophages, neutrophils, and eosinophils were present in the lungs of uninfected *Cse* ^−/−^ mice *versus* WT (Rahman et al., 2020). However, these differences were reversed after *Mtb* infection, when significantly more alveolar macrophages, neutrophils, and eosinophils were observed in WT lungs compared to those of *Cse* ^−/−^ mice (**Figure 5**). These observations suggested that CSE in the WT infected mice promotes an excessive innate immune response, which is consistent with other TB studies that show increased neutrophils can exacerbate TB disease (Nandi and Behar, 2011; Lowe et al., 2012) and that H_2_S can initiate neutrophil infiltration during *Mtb* infection (Rahman et al., 2020; Saini et al., 2020) and septic shock (Ang et al., 2011b).

#### H_2_S and Regulation of Adaptive Immunity

The lungs of uninfected *Cse^−^* ^/−^ mice had more CD8^+^ T cells, but not CD4^+^ T cells, compared to WT mice (Rahman et al., 2020). At 2- and 6-weeks post-infection, the number of CD4^+^ and CD8^+^ T cells and their effector memory populations (CD62L^lo^CD44^hi^) in the lungs were higher in *Cse* ^−/−^ mice than in WT (**Figure 5**). Hence, the presence of CSE leads to suppressed adaptive immune response to *Mtb* infection. The protective role of innate and adaptive immunity to control *Mtb* infection has been discussed in several reviews (Mogues et al., 2001; Zuniga et al., 2012; O’Garra et al., 2013; Sia and Rengarajan, 2019). Further, examination of regulatory T cells (Tregs, CD4^+^CD25^+^) in uninfected mice revealed more cells in WT compared to *Cse* ^−/−^ mice, and no differences in the number of CD8^+^ Tregs. However, after *Mtb* infection, the number of CD25^+^FoxP3^−^ cells was increased in *Cse* ^−/−^ mice compared to WT in both CD4^+^ and CD8^+^ T populations. The number of CD4^+^CD25^+^FoxP3^+^ Tregs was lower in *Cse* ^−/−^ mice lungs at 2 weeks post-infection compared to WT, which was reversed after 4 weeks of infection, suggesting that *Cse* ^−/−^ mice can better control pro-inflammatory immune responses at later time points. While a strong immune response resulting from increased innate and adaptive immune cells may be required to control *Mtb* infection and disease progression, excessive inflammation is detrimental to the host at later stages of infection (Cardona and Cardona, 2019; Okeke and Uzonna, 2019). Tregs are important for controlling pathogen-induced inflammatory responses such as neutrophil activity and T cell proliferation caused by both innate and adaptive immune cells (Cardona and Cardona, 2019; Okeke and Uzonna, 2019). Therefore, a balance between immunosuppressive Tregs and effector T cells, in this case Th1 and Th17 responses against TB, is necessary to simultaneously control *Mtb* growth and protect host tissue from inflammation-mediated damage (Cardona and Cardona, 2019; Okeke and Uzonna, 2019). Interestingly, in CD4^+^ and CD8^+^ T cell populations, the number of IFN-γ^+^ cells was greater in uninfected WT mice. However, this was reversed upon *Mtb* infection where *Cse* ^−/−^ mice had significantly more CD4^+^IFN-γ^+^ and CD8^+^IFN-γ^+^ T cells than the WT (**Figure 5**). Increased numbers of IFN-γ–producing T (Th1) cells in *Cse* ^−/−^ mice were reflected by the increased number of effector memory T cells, decreased levels of neutrophils (Nandi and Behar, 2011; Rahman et al., 2020), and the subsequent control of *Mtb* growth *in vivo* (Rahman et al., 2020).

#### H_2_S and Serum Cytokines

Measurement of serum cytokine levels revealed that *Cse* ^−/−^ mice had elevated levels of pro-inflammatory cytokines IL-1α, IL-1β, IL-6, TNF-α, IL-12, IFN-γ, and IL-17, well known for controlling *Mtb* infection (Zuniga et al., 2012; O’Garra et al., 2013; Domingo-Gonzalez et al., 2016; Sia and Rengarajan, 2019) at 2 or 3 weeks after *Mtb* infection, compared to WT controls (Rahman et al., 2020). Further, *Cse* ^−/−^ mice had lower levels of anti-inflammatory cytokines IL-10 and IL-13 at 2 weeks post-infection, which was followed by a significant increase in IL-10 levels after 3 weeks of infection. These data suggest precise control of the pro-inflammatory response in *Cse* ^−/−^ mice. Similarly, elevated levels of pro-inflammatory cytokines TNF-α, IL-1α, IL-6, IL-12, IL-17, and IFN-γ were observed in the serum of *Mtb*-infected *Cbs* ^+/−^ mice compared to WT mice (Saini et al., 2020). The efficient induction of Th1 immunity is critical for defense against *Mtb*. Understandably, defects in Th1 cytokine (IFN-γ) production are established risk factors for *Mtb* infection and disease progression in humans and mice (Rossouw et al., 2003; O’Garra et al., 2013; Sia and Rengarajan, 2019). While IFN-γ is pro-inflammatory at the onset of *Mtb* infection and critical for macrophage activation, it also exerts homeostatic functions that help minimize inflammation-mediated lung damage by limiting neutrophil accumulation (Hu and Ivashkiv, 2009; Nandi and Behar, 2011). Mice that lack IFN-γ are extremely susceptible to *Mtb* infection due primarily to the inability to activate macrophages, and form large necrotic pulmonary lesions within weeks of *Mtb* infection (Cooper et al., 1993; Pearl et al., 2001). In agreement with the role of IFN-γ in limiting neutrophil recruitment, increased CSE and CBS levels (which correlate with reduced IFNγ) were associated with neutrophil accumulation and inflammation in infected lungs, leading to progressive *Mtb* growth and disease progression (Rahman et al., 2020; Saini et al., 2020). However, *Cse* ^−/−^ and *Cbs* ^+/−^ mice are more efficient in controlling *Mtb* infection due to an increased adaptive response, particularly IFN-γ and IL-17 production (Rahman et al., 2020; Saini et al., 2020). In sum, upregulation of the H_2_S producing enzymes CSE and CBS in TB is associated with reduced production of pro-inflammatory cytokines and increased levels of anti-inflammatory cytokines, consistent with increased bacterial growth and more severe pathology (**Figure 5**).

### H_2_S Promotes *Mtb* Growth by Suppressing Pro-Inflammatory Cytokines

**Figure 6:**
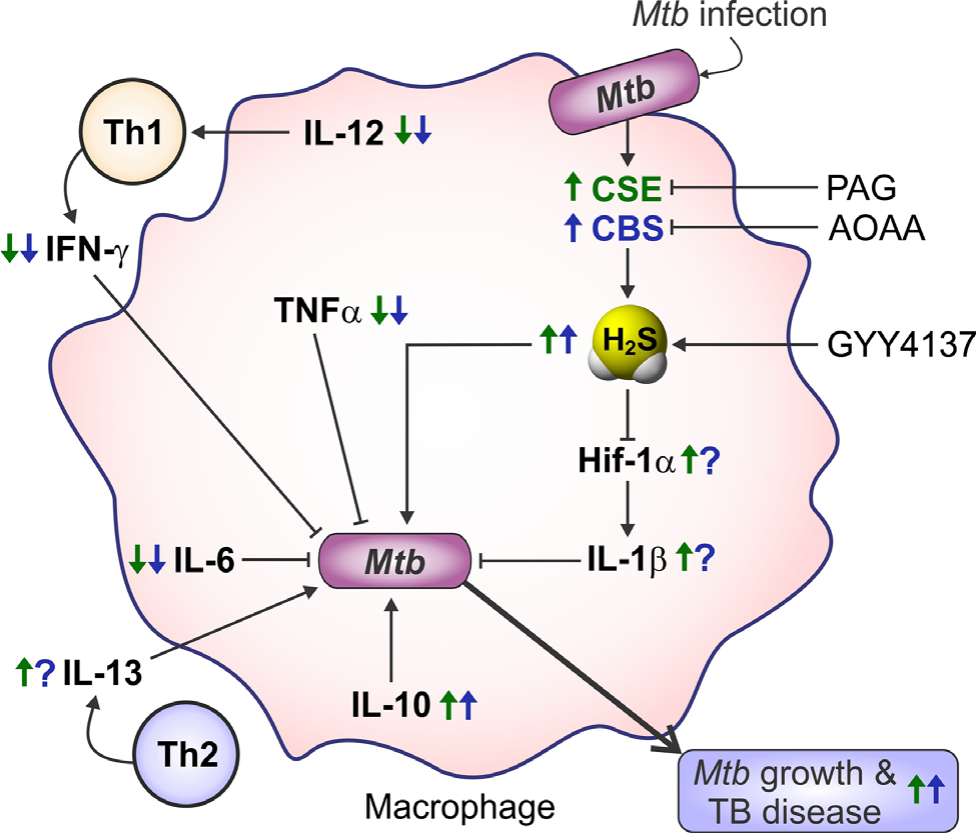
Immune regulation by H_2_S in *Mtb*-infected macrophages. *Mtb* infection increases H_2_S levels *via* increased expression of CSE and CBS. Hif-1α promotes the production of pro-inflammatory cytokines IL-1β, TNF-α, IL-6, and IL-12 which are critical for limiting *Mtb* growth. CSE/H_2_S reduces Hif-1α levels, thereby reducing the level of pro-inflammatory cytokines, and upregulates the anti-inflammatory Th2 cytokines IL-10 and IL-13, which support intracellular *Mtb* growth and exacerbate TB pathogenesis. PAG—a specific inhibitor of CSE; AOAA—CBS inhibitor; GYY4137—a slow releaser of H_2_S. Blue and green arrows indicate the molecular regulation caused by CBS and CSE, respectively. Arrows indicate an H_2_S-mediated increase (up) or decrease (down) in the activity associated with TB disease. Question marks (?) indicate that the role of CBS is unknown.

In the context of *Mtb* infection, H_2_S was shown to be the CSE- and CBS-generated effector molecule in murine peritoneal macrophages. *Mtb* infection of murine macrophages led to increased expression of CBS as early as 6 h post-infection (Saini et al., 2020). Similarly, increased CSE and CBS expression was observed 24 h after *Mtb* infection of macrophages (Rahman et al., 2020), which directly contributed to increased H_2_S production. Cysteine is used by CSE and CBS to produce H_2_S. Increasing the cysteine concentration in the culture media of murine macrophages increased H_2_S production, whereas addition of PAG reduced H_2_S production. Consistent with this, *Mtb* growth was reduced in *Cse* ^−/−^ and *Cbs* ^+/−^ macrophages compared to WT controls. Addition of GYY4137 to *Cse* ^−/−^ and *Cbs* ^+/−^ macrophages promoted *Mtb* growth to the level of WT, whereas addition of PAG or AOAA to WT macrophages reduced *Mtb* growth to the level of *Cse^−^* ^/−^ and *Cbs* ^+/−^ macrophages (**Figure 6**) (Rahman et al., 2020; Saini et al., 2020). These data indicate that H_2_S is the CBS- and CSE-related effector responsible for *Mtb* growth. Also, production of pro-inflammatory cytokines IL-1β and IL-6 was higher in *Mtb*-infected *Cse* ^−/−^ macrophages compared to WT, which was further decreased by treatment with GYY4137. *Mtb* infection of *Cse* ^−/−^ macrophages significantly increased the percentage of IL-12-positive cells compared to WT. This result was notable since IL-12 links the innate to adaptive immune responses and is involved in the differentiation of Th1 cells for IFN-γ production to control *Mtb* growth and its dissemination (Cooper et al., 1993; Cooper et al., 1997; Sakai et al., 2016).

### Host H_2_S Suppresses Glycolysis and Oxygen Consumption in Macrophages

**Figure 7:**
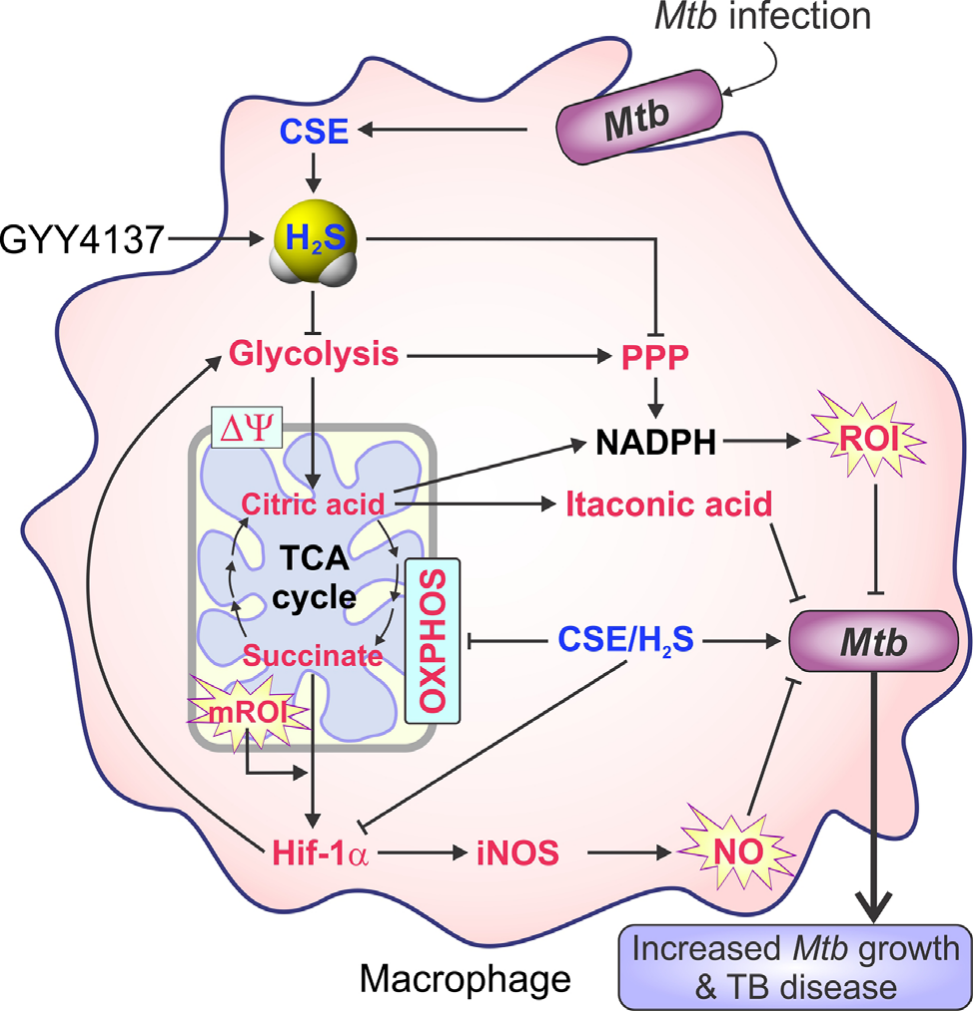
H_2_S reprograms carbon metabolism in *Mtb*-infected macrophages. *Mtb* infection increases H_2_S production, which suppresses Hif-1α expression followed by reduced glycolytic and PPP, TCA cycle, and oxidative phosphorylation (OXPHOS) activity. Apart from regulating inflammation, Hif-1α also increases the expression of glycolytic enzymes. Hence, Hif-1α links metabolic and immune processes. Molecules in blue text are upregulated, and those in red text are downregulated in *Mtb*-infected murine macrophages.

H_2_S is known to modulate glycolysis and mitochondrial respiration (Fu et al., 2012; Modis et al., 2014; Szabo et al., 2014; Liang et al., 2015). The role of host-derived H_2_S in macrophage energy metabolism was determined using an Agilent Seahorse XF96 flux analyzer to measure the extracellular acidification rate (ECAR) to examine glycolytic functions, and the oxygen consumption rate (OCR) to examine OXPHOS of macrophages (Cumming et al., 2018; Russell et al., 2019). *Mtb*-infected *Cse* ^−/−^ macrophages exhibited increased glycolysis and a higher glycolytic capacity than WT macrophages (Rahman et al., 2020). These observations are consistent with the notion that H_2_S production in WT macrophages decelerates glycolysis, making them less capable of controlling *Mtb* growth and disease progression, which is consistent with recent studies (Cumming et al., 2018; Hackett et al., 2020). Further, OCR, an indicator of cellular respiration, was measured in WT and *Cse* ^−/−^ macrophages. The OCR, basal respiration, and ATP production were increased in uninfected and *Mtb*-infected *Cse* ^−/−^ macrophages compared to WT controls. Of note, addition of GYY4137 to *Cse* ^−/−^ macrophages reduced the OCR for basal respiration, ATP production, and non-mitochondrial respiration to WT levels. Conversely, addition of the CSE inhibitor PAG to infected WT macrophages increased the basal respiration and non-mitochondrial respiration similar to that of *Cse* ^−/−^ cells. These data clearly show that CSE-generated H_2_S can modulate energy metabolism in macrophages by suppressing glycolysis and mitochondrial respiration (**Figure 7**), which can alter immune cell function (Rahman et al., 2020).

### H_2_S Suppresses Central Carbon Metabolism in Macrophages

The bioenergetic differences observed between WT and *Cse* ^−/−^ macrophages suggest that H_2_S is a regulator of energy metabolism in macrophages. To determine the role of CSE in central carbon metabolism, WT and *Cse* ^−/−^ macrophages were infected with *Mtb*, with or without GYY4137, and energy-related metabolites were quantified by liquid chromatography–mass spectrometry (LC-MS/MS). This study suggested that excessive H_2_S produced by upregulation of CSE following *Mtb* infection suppresses central carbon metabolism (Rahman et al., 2020). For example, while glycolytic metabolites (fructose 6-phosphate, fructose 1,6-bisphosphate, dihydroxyacetone phosphate, phosphoenolpyruvate, pyruvate, and lactate) in uninfected *Cse* ^−/−^ macrophages were moderately increased compared to WT, *Mtb* infection markedly increased glycolytic metabolites in *Cse* ^−/−^ macrophages. However, addition of GYY4137 to *Cse* ^−/−^ macrophages reduced the levels of these metabolites to that of WT macrophages. Further, a dysregulated TCA cycle with higher levels of citrate and succinate was observed in *Cse* ^−/−^ macrophages, which was restored upon *Mtb* infection. Lastly, increased levels of α-ketoglutarate and succinate were present in *Mtb*-infected *Cse* ^−/−^ macrophages, but GYY4137 treatment brought these metabolites down to the level of WT macrophages.

High levels of succinate stabilize HIF-1α, leading to increased expression of IL-1β, a cytokine essential for controlling *Mtb* growth (Jayaraman et al., 2013; Tannahill et al., 2013). Interestingly, the metabolic phenotype observed in *Cse* ^−/−^ macrophages is characteristic of the metabolic rewiring that occurs in pro-inflammatory macrophages (O’Neill et al., 2016), suggesting that uninfected *Cse* ^−/−^ macrophages are polarized toward a pro-inflammatory phenotype prior to infection. In addition, levels of metabolites in the pentose phosphate pathway (PPP) (ribulose-5-phosphate, sedoheptulose-7-phosphate, and erythrose-4-phosphate) were significantly increased in *Mtb*-infected *Cse* ^−/−^ macrophages compared to WT. These metabolic differences suggest that increased H_2_S production in macrophages following *Mtb* infection suppresses the PPP (**Figure 7**). Moreover, increased levels of Hif-1α were observed in *Mtb*-infected *Cse* ^−/−^ macrophages compared to WT controls. Of note, Hif-1α is essential to promote glycolysis and production of pro-inflammatory cytokines like IL-1β, IL-6, IL-12, and IFN-γ (Braverman et al., 2016; Gleeson and Sheedy, 2016; O’Neill et al., 2016; Marks et al., 2017; Wang T. et al., 2017). In different studies, exogenous H_2_S was shown to increase Hif-1α accumulation under normoxia in *Caenorhabditis elegans* (Budde and Roth, 2010), or lower Hif-1α levels during hypoxia in cultured cells (Budde and Roth, 2010; Kai et al., 2012; Wu et al., 2012). Overexpression of CSE in HEK293T cells resulted in reduced Hif-1α protein accumulation under hypoxic conditions, whereas under normoxic conditions, addition of 100 μM NaHS to cultures of Hif-1α-overexpressing HEK293T cells also significantly reduced Hif-1α protein levels *via* inhibition of Hif-1α protein translation (Wu et al., 2012). This indicates that endogenous or exogenous H_2_S reduces Hif-1α expression irrespective of oxygen tension (Wu et al., 2012). Consistent with this finding, *Cse* ^−/−^ cells showed increased Hif-1α levels after *Mtb* infection compared to WT cells (Rahman et al., 2020). Overall, these findings suggest that excessive H_2_S produced by *Mtb*-infected macrophages suppresses glycolysis and triggers a reduction in Hif-1α levels causing reduced production of pro-inflammatory cytokines and increased bacterial growth.

### CSE Suppresses Mitochondrial and Cellular Oxidative Stress

Studies in mice have shown that inhalation of H_2_S at 80 parts per million reduces inflammation and the formation of reactive oxygen intermediates (ROI) that occurs during ventilator-induced lung injury (Spassov et al., 2017). Since mitochondrial respiration is subject to regulation by H_2_S, a role for H_2_S in modulating mitochondrial mass, mitochondrial membrane potential (Δψ_m_), and production of mitochondrial reactive oxygen intermediates (mROI) was investigated during *Mtb* infection. *Mtb* infection of WT mouse macrophages significantly decreased mitochondrial mass; however, no change was observed in *Cse* ^−/−^ macrophages (Rahman et al., 2020). Measurement of Δψ_m_ revealed that following *Mtb* infection, overall Δψ_m_ was decreased in both *Cse* ^−/−^ and WT macrophages. However, *Cse* ^−/−^ macrophages retained a more polarized Δψ_m_ than did WT cells. The observed decrease in Δψ_m_ in WT macrophages after *Mtb* infection is consistent with their lower basal respiration compared to *Cse* ^−/−^ macrophages. Quantitation of mROI revealed that mROI levels in uninfected WT and *Cse* ^−/−^ macrophages were similar; however, *Mtb* infection lowered mROI levels only in WT cells. Further, cellular reactive nitrogen intermediates (RNI) and cellular ROI were measured. While *Mtb* infection increased RNI levels in both *Cse* ^−/−^ and WT macrophages, RNI levels in *Cse* ^−/−^ macrophages were consistently higher than in WT macrophages irrespective of infection. The presence of increased RNI/nitric oxide in *Cse* ^−/−^ macrophages is consistent with higher IFN-γ levels seen in *Cse* ^−/−^ macrophages, which can limit *Mtb* growth. Several studies have demonstrated that at physiological concentrations, H_2_S can function as an antioxidant by reducing the formation of mROI and RNI (Predmore et al., 2012; Modis et al., 2014; Corsello et al., 2018; Shefa et al., 2018; Xiao et al., 2018). Consistent with this finding, *Cse* ^−/−^ macrophages produce more mROI and cellular RNI following *Mtb* infection (**Figure 7**) (Rahman et al., 2020).

### H_2_S and M2 Macrophage Polarization in Tuberculosis

As mentioned above in *Role of H_2_S in Macrophage Polarization*, considerable evidence indicates that H_2_S is involved in macrophage polarization and can drive macrophages toward an anti-inflammatory M2 phenotype (**Figure 4**). An anti-inflammatory role for H_2_S was also observed in a recent study that examined the role of H_2_S in TB using *Cse* ^−/−^ mice and macrophages. For example, infected *Cse* ^−/−^ macrophages, which produce less H_2_S than infected WT cells, exhibit increased pro-inflammatory cytokine production, increased glycolysis, and flux through the PPP, and higher levels of mROI, iNOS (*Nos2*), RNI, and Hif-1α expression compared to WT macrophages. This functional profile provided more efficient control of *Mtb* growth and disease compared to WT macrophages. In contrast, *Mtb*-infected WT macrophages (which exhibited the highest H_2_S production) showed increased production of anti-inflammatory cytokines IL-10 and IL-13, and arginase 1/2 (*Arg1/2*) compared to *Cse* ^−/−^ macrophages. Notably, chemical complementation of *Mtb*-infected *Cse* ^−/−^ macrophages with GYY4137 produced a functional profile similar to WT cells, characterized by a reduction in pro-inflammatory cytokines IL-1β and IL-6 and reduced levels of glycolytic and PPP intermediates. Overall, these findings indicate that the CSE/H_2_S axis is a critical determinant of macrophage phenotype in TB disease, and that increased CSE/H_2_S levels appear to promote M2 type polarization and enhanced TB control (**Figure 4**) (Rahman et al., 2020).

### H_2_S Stimulates *Mtb* Growth and Metabolism

**Figure 8:**
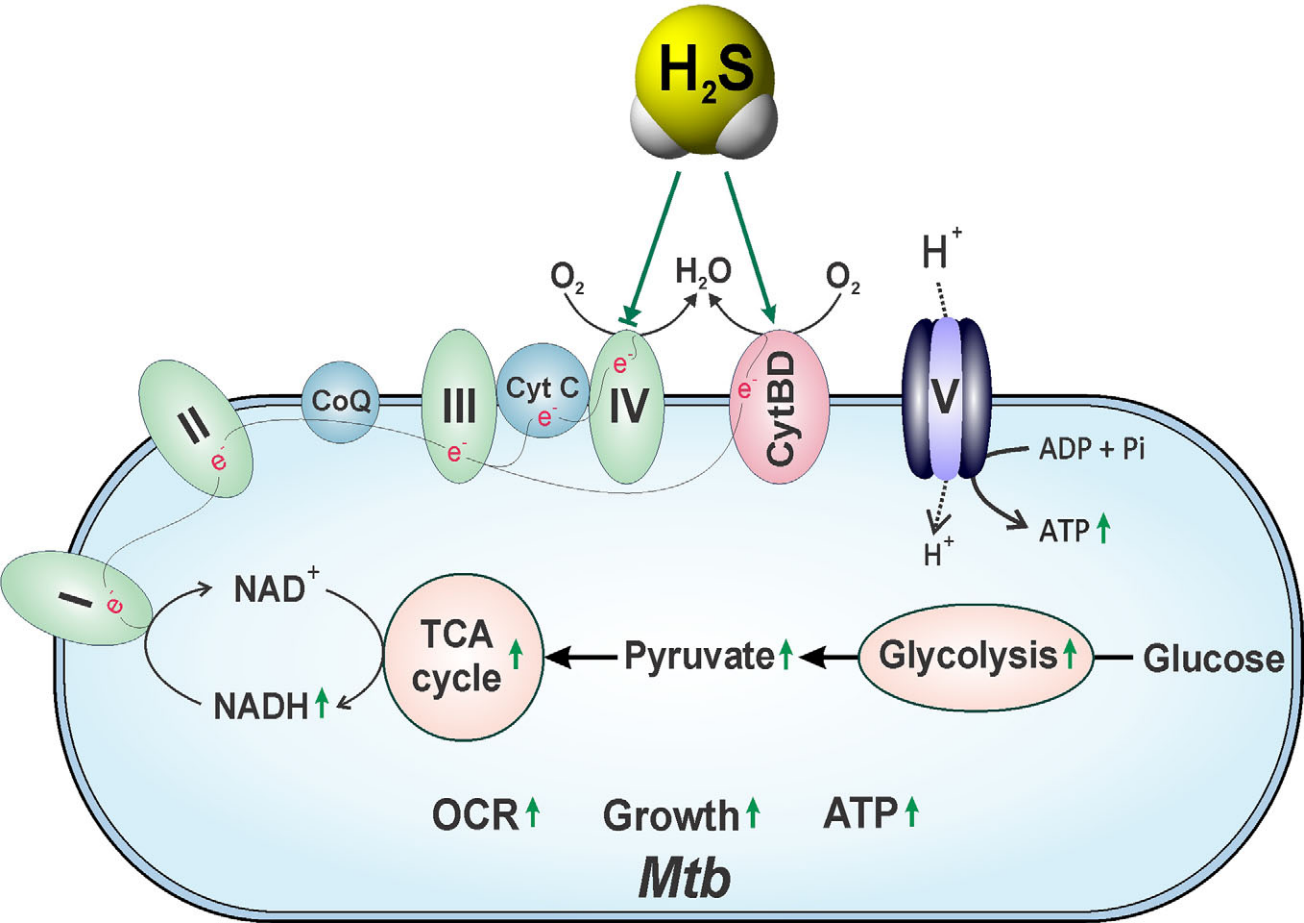
The roles of host-generated H_2_S in *Mtb* respiration and growth. Host-generated H_2_S can stimulate *Mtb* respiration and bioenergetics predominantly through cytochrome *bd* oxidase CytBD, thereby increasing bacterial growth and accelerating disease.

H_2_S is membrane-permeable; therefore, host-derived H_2_S can influence *Mtb* residing in host cells. Evidence that H_2_S directly modulates *Mtb* growth was provided by addition of GYY4137, a slow releaser of H_2_S, to mimic host-derived H_2_S. Addition of low concentrations of GYY4137 (5–25 μM) to *Mtb* cultures stimulated *Mtb* growth; however, at higher concentrations growth stimulation was lost, demonstrating a bimodal effect (Saini et al., 2020). Consistent with these observations, *Mtb* growth was attenuated in *Cbs* ^+/−^ and *Cse* ^−/−^ macrophages, compared to WT, consistent with reduced organ burden and increased survival observed in these mice (Saini et al., 2020; Rahman et al., 2020). Further, addition of GYY4137 to *Mtb*-infected *Cbs* ^+/−^ and *Cse* ^−/−^ macrophages increased *Mtb* proliferation *versus* untreated controls, and addition of the CBS or CSE inhibitors to infected WT macrophages reduced *Mtb* CFUs to levels seen in *Cbs* ^+/−^ or *Cse* ^−/−^ macrophages respectively (**Figure 6**). H_2_S-mediated growth stimulation of *Mtb* was accompanied by an increased oxygen consumption rate (OCR) and increased intracellular ATP. Further, high-resolution metabolite studies using LC-MS/MS showed that H_2_S exposure also led to increased levels of glycolytic and TCA cycle metabolic intermediates. The H_2_S-mediated stimulation of respiration was found to be largely dependent on cytochrome *bd*-type quinol oxidase (CytBD), as *Mtb* mutants defective in cytochrome *bd* oxidase activity showed highly attenuated growth and bioenergetic responses to H_2_S (Saini et al., 2020). This CytBD-mediated stimulation of respiration in *Mtb* is consistent with data showing that CytBD in *Escherichia coli* is resistant to H_2_S-mediated inhibition of respiration (Forte et al., 2016; Korshunov et al., 2016). Further, H_2_S was shown to alter gene expression in *Mtb*. Upon addition of GYY4137, transcriptomic analysis showed upregulation of genes belonging to the DosR/S/T dormancy regulon, CsoR and RicR copper regulons, and several genes involved in sulfur metabolism. In line with these data, H_2_S supports *Mtb* entry into *in vitro* dormancy and recovery from dormancy after reaeration. In addition, *Mtb* exposed to H_2_S exhibits enhanced growth under conditions of oxidative stress compared to untreated *Mtb*, suggesting an antioxidant function for H_2_S (Saini et al., 2020). Further, a role for H_2_S in redox balance and antibiotic tolerance in *Mtb* has been reported (Mishra et al., 2019). Overall, it is reasonable to propose that the presence of H_2_S at the site of infection can support the growth of *Mtb* by rewiring central carbon metabolism and improving the bioenergetic health of *Mtb* (**Figure 8**).

## Role of Endogenous H_2_S in Bacterial Physiology

While the focus of this review is to highlight the diverse functions of host-derived H_2_S in TB and other microbial diseases, a brief overview of the physiological relevance of bacterially-derived H_2_S is warranted. Unlike H_2_S in mammals, the production of H_2_S by bacteria has been known for over 140 years, beginning with the work of French microbiologist Ulysse Gayon in 1877; however, the role of H_2_S as an important effector molecule in bacterial physiology was not appreciated until the 1960s (Szabo, 2018). Orthologs of mammalian H_2_S-producing enzymes CBS, CSE, and/or 3-MST are present in most bacterial species, indicating their importance and evolutionary conservation. For example, CBS and CSE activity is the main source of H_2_S production in *Klebsiella pneumoniae*, *Bacillus anthracis*, *Pseudomonas aeruginosa*, and *Staphylococcus aureus*, whereas 3-MST is the primary source of H_2_S production in *Escherichia coli* (Shatalin et al., 2011; Mironov et al., 2017). Consistent with the presence of bacterial orthologs of CSE and CBS, L-cysteine is also a common substrate for H_2_S production in many bacteria. Addition of L-cysteine has been shown to stimulate H_2_S production in *B. anthracis*, *P. aeruginosa*, *S. aureus*, and *E. coli* (Shatalin et al., 2011; Mironov et al., 2017).

Recent studies have identified specific physiological functions of endogenous H_2_S in bacteria. A major finding was that H_2_S can protect bacteria from antibiotics and oxidative stress. Inactivation of CBS and CSE in *B. anthracis*, *P. aeruginosa*, and *S. aureus* or 3-MST in *E. coli* *via* genetic deletion or enzymatic inhibitors reduced H_2_S production by 90% and rendered these pathogens susceptible to gentamycin, ampicillin, and nalidixic acid compared to untreated or WT controls (Shatalin et al., 2011). Further, 3-MST overproduction in *E. coli* provides increased resistance to spectinomycin. Exogenous H_2_S (200 μM NaHS) suppressed antibiotic sensitivity in CBS/CSE-double knockout or 3-MST-deficient bacteria. *E. coli* exhibited increased resistance to gentamycin when cultured in cysteine-rich (0.5 mM) media compared to standard media (<20 μM cysteine) (Shatalin et al., 2011). In contrast, a 3-MST-mutant of *E. coli* is more sensitive to gentamycin in cysteine-rich media, and was protected by thiol-depleting diamide, suggesting that pro-oxidative cysteine accumulates in this mutant. H_2_S-deficient bacteria were highly susceptible to peroxide, whereas NaHS protects them from peroxide-generated oxidative stress, indicating that H_2_S can increase the antioxidant capacity of bacterial cells. In conclusion, H_2_S provides antibiotic resistance to clinically relevant and evolutionarily distant pathogenic bacteria by alleviating oxidative stress (Shatalin et al., 2011). Another study demonstrated that endogenous H_2_S produced by 3-MST protects *E. coli* from oxidative stress and genome damage by lowering cysteine levels and sequestering free iron that drives genotoxic Fenton chemistry that generates hydroxyl radicals (Mironov et al., 2017). Further, 3-MST-derived H_2_S plays a role in regulating levels of intracellular cysteine, which can be toxic above physiological levels (Mironov et al., 2017). Other reports have shown that exogenous H_2_S helps *E. coli* to maintain redox homeostasis and protects bacteria against antibiotic-triggered oxidative stress. Inhibition of H_2_S biosynthesis reversed antibiotic resistance in multidrug-resistant uropathogenic *E. coli*, whereas exposure to a bacteria-specific, enzyme-activated H_2_S donor compound restored drug tolerance (Shukla et al., 2017).

In contrast, a study on *Acinetobactor baumannii*, a pathogenic bacterium that cannot produce H_2_S, demonstrated that treatment with exogenous H_2_S (80–160 μM NaHS) conferred hypersensitivity to various antibiotics (Ng et al., 2020). In fact, exogenous H_2_S in *A. baumannii* potentiated the killing effect of antibiotics including gentamycin, colistin, rifampicin, and clarithromycin. Moreover, exposure of a gentamycin-resistant clinical isolate of *A. baumannii* to NaHS reverted the resistance to gentamycin. H_2_S exacerbated antibiotic-mediated killing by increasing the Fe^2+^/Fe^3+^ ratio (Fe^2+^ mediates ROI generation *via* Fenton chemistry), increasing ROI, and by depolarizing the membrane potential which caused a reduction in ATP levels (Ng et al., 2020).

*E. coli* exposed to 0.5 mM L-cysteine/L-cystine exhibited a transient increased susceptibility to H_2_O_2_ resulting in an unusually rapid rate of DNA damage. However, treatment with iron chelators (dipyridyl or desferrioxamine) eliminated this sensitivity, indicating that intracellular free iron mediates the conversion of H_2_O_2_ into hydroxyl radicals, the effector of DNA damage (Park and Imlay, 2003). A higher cysteine concentration (10 mM) promoted bacterial respiration and ROI production, and potentiated the killing effect of ampicillin, kanamycin, and ciprofloxacin against bacterial persisters (*E. coli*, *Salmonella enteritidis*, *A. baumannii*, and *P. aeruginosa*) in the stationary phase, but not in the exponential growth phase, indicating that the synergistic killing effect of cysteine is dependent on the metabolic state of the bacterium (Liu et al., 2020).

Of note, *Mtb* encodes putative orthologs of CBS (Rv1077), CSE (Rv1079), and 3-MST (Rv2291). Biochemical studies of the *Mtb* transsulfuration pathway revealed that Rv1079 is a dual-function enzyme with CSE activity (catalyzes cystathionine to cysteine) and cystathionine γ-synthase (CGS) activity (catalyzes O-succinylhomoserine and cysteine to cystathionine) (Wheeler et al., 2005). Interestingly, Rv1079 (CSE/CGS) does not have cysteine desulfhydrase (CDS) activity (CDS converts cysteine to pyruvate, NH_3_, and H_2_S) unlike CSE in other organisms including mammals. CDS activity has been observed in WT *Mtb* as well as a *Mtb* *rv1079* deletion mutant, and this activity was not inhibited by PAG. Hence, the CSD activity in *Mtb* is catalyzed by an unknown protein(s) using L-cysteine or L-cystine as a substrate (Wheeler et al., 2005). A study showed that the addition of cysteine or other small thiols to either isoniazid (INH) or rifampicin prevents the formation of drug-tolerant and drug-resistant cells in *Mtb* cultures in a concentration- (8 μM–4 mM) and time-dependent manner (Vilcheze et al., 2017). Moreover, the combination of 4 mM cysteine and INH (7.3 μM, 20 x MIC) sterilized *Mtb* cultures. The increased killing of INH/cysteine-treated *Mtb* cultures resulted from increased cellular respiration, ferrous (Fe^2+^) ion and ROI leading to oxidative stress and DNA damage (Vilcheze et al., 2017). However, this study did not consider a possible role for H_2_S arising from the CDS activity in *Mtb* (Wheeler, et al., 2005), which may influence the effects of antibiotics on *Mtb*.

**Figure 9:**
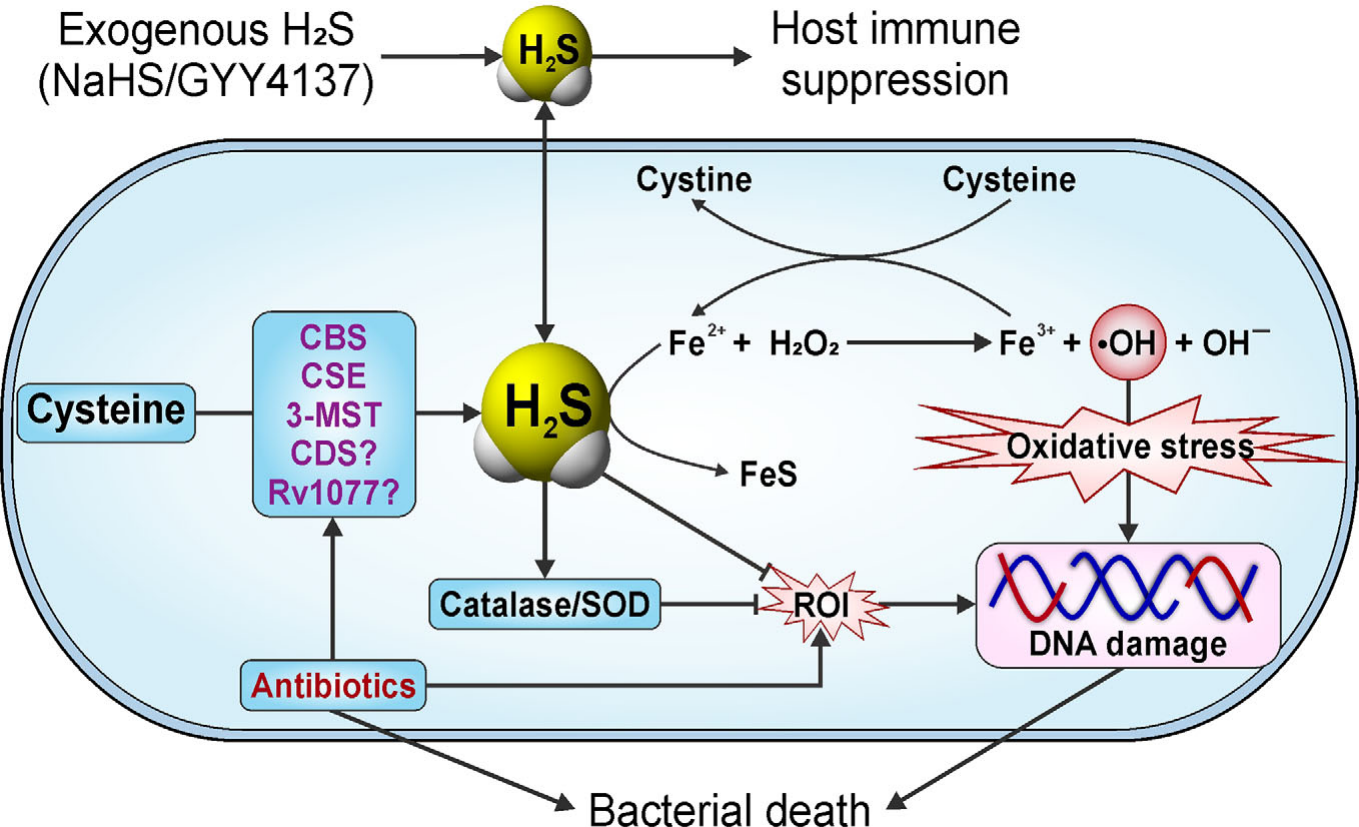
Endogenously produced H_2_S protects bacteria against oxidative stress. Endogenously produced or exogenous H_2_S protects bacteria from oxidative stress triggered by antibiotics and/or host against infection. This protection is achieved by multiple mechanisms: i) increased activity of H_2_S producing enzymes; ii) depletion of excess cysteine (cysteine generates Fe^2+^ for Fenton chemistry); iii) inhibition of the Fenton reaction, since H_2_S reacts with H_2_O_2_ and free Fe^2+^); iv) increased activity of catalase and superoxide dismutase (SOD); v) suppression of host immune responses. Question marks (?) indicate that the functions of CDS and Rv1077 in *Mtb* are unknown.

Finally, to gain insight into the role of bacterial H_2_S in defense against host immunity, a study using *E. coli* and *S. aureus*, common sources of nosocomial infections, showed that endogenous H_2_S provides resistance against immune-mediated killing (Toliver-Kinsky et al., 2019). This was demonstrated by several observations—1) decreased bacterial killing by mouse leucocytes, when infected with bacteria treated with GYY4137 (0.3 and 1 mM), 2) bacterial clearance from leucocytes or RAW264.7 cells was significantly increased after inhibiting 3-MST in *E. coli* or CBS/CSE in *S. aureus* using either an inhibitor or a gene deletion mutant, and 3) Mice infected with H_2_S-deficient bacteria had a lower burden in the spleen and decreased plasma levels of IL-6 compared to infection with WT bacteria (Toliver-Kinsky et al., 2019). In conclusion, endogenous H_2_S in bacteria can suppress host immunity and reduce oxidative stress triggered by antibiotics (**Figure 9**).

## Conclusion and Future Directions

This review critically summarizes the wide-ranging physiological roles of H_2_S in mammals and highlights how host-generated H_2_S impacts *Mtb* growth, disease progression, and immunometabolism in TB. Despite the documented role of H_2_S in numerous pathophysiological processes, few studies have attempted to elucidate the role of host-derived H_2_S in the control of microbial pathogens. The lack of data concerning the intersection of host-derived H_2_S and infectious disease provides the research community with unique opportunities to make new, innovative contributions to the study of bacterial and viral pathogens. Given the numerous studies that followed the discovery of iNOS (*Nos2*) and the importance of •NO bioactivity in microbial pathogenesis (MacMicking et al., 1997b; Das et al., 2010), we anticipate a rapid increase in studies that further define the importance of H_2_S in microbial pathogenesis.

Within the context of TB, several previously unexplored areas of interest have been identified. Firstly, studies have clearly shown that H_2_S-deficient *Cse* ^−/−^ and *Cbs* ^+/-^ mice have less severe TB disease (Rahman et al., 2020; Saini et al., 2020). This is intriguing, because to the best of our knowledge, the mortality of other knockout mice infected with *Mtb* has been unchanged or increased compared to wild-type controls. These findings suggest that CBS or CSE are suitable targets for host-directed therapeutic intervention in TB. Since *Mtb* infection induces excessive production of H_2_S, which is sensed by *Mtb* *in vivo* to promote growth (Saini et al., 2020), and which suppresses host glycolysis (Rahman et al., 2020), it will be important to identify the underlying immunometabolic mechanisms that contribute to disease. For example, how does excessive H_2_S production following *Mtb* infection regulate immunity, and how does H_2_S suppress glycolysis in host cells upon *Mtb* infection? Secondly, several other lines of investigation may help establish new virulence paradigms. For example, recent reports showing that *Mtb* suppresses host glycolysis (Cumming et al., 2018; Hackett et al., 2020) are consistent with the demonstration that H_2_S is the effector molecule that suppresses glycolysis (Rahman et al., 2020) and with studies showing that H_2_S targets glycolytic enzymes (Guo et al., 2019). Thirdly, while the TB field has benefited greatly from several animal models of TB, no single animal model represents the full histopathological spectrum of human pulmonary TB (Hunter, 2016). Therefore, it is essential that we increase our understanding of the role of H_2_S-producing enzymes in human pulmonary TB. For example, using flow cytometry, immunohistochemistry, or other novel “-omic” technologies to examine the cellular distribution of H_2_S-producing enzymes within cavities, necrotic and non-necrotic granulomas of human pulmonary TB (Chinta et al., 2018; Reddy et al., 2018; Rahman et al., 2020) will make important contributions to the field. Although this has been accomplished to a limited extent by a previous study (Rahman et al., 2020), comprehensive characterization of human TB tissues is needed. Also, it is tempting to speculate that exhaled H_2_S could be used as a volatile biomarker for rapid diagnosis of TB. Fourthly, there is compelling evidence that host-derived H_2_S is sensed by *Mtb* to reprogram its metabolism and stimulate growth (Saini et al., 2020). Outstanding questions include: by what mechanism is exogenous H_2_S sensed by *Mtb*? How does host-derived H_2_S reprogram *Mtb* metabolism to promote virulence and growth? And lastly, how does host-derived H_2_S modulate *Mtb* dormancy? Answers to these questions are likely to reveal new insights and establish new paradigms whereby *Mtb* causes disease. Fifthly, it is difficult to ignore the obvious link between H_2_S and •NO, as these two gasotransmitters chemically interact with each other (Kolluru et al., 2013) and could possibly regulate the heme prosthetic groups of CBS and iNOS. Of note, CBS- or CSE-generated H_2_S and iNOS-generated •NO differ substantially in their ability to alter the course of *Mtb* infection; whereas mice lacking *Nos2* are more susceptible to *Mtb* infection (MacMicking et al., 1997b), *Cse* ^−/−^ and *Cbs* ^+/-^ mice are more resistant to *Mtb* infection (Rahman et al., 2020; Saini et al., 2020). Hence, it will be important to understand the role of H_2_S in *Nos2*-deficient mice, and the role of •NO in *Cse* ^−/−^ and *Cbs* ^+/-^ mice.

In conclusion, a fundamental challenge in the TB field is to improve our understanding of the mechanisms whereby *Mtb* causes disease. The gasotransmitter H_2_S plays an unusual role in the control of TB and provides new knowledge which could be exploited for successful TB intervention strategies.

## Author Contributions

All authors contributed to the article and approved the submitted version.

## Funding

This work was supported by NIH Grants R01AI134810, R01AI137043, R01AI152110, R33AI138280, a Bill and Melinda Gates Foundation Award (OPP1130017), the South African Medical Research Council, and an NRF BRICS Multilateral grant to AJCS.

## Conflict of Interest

The authors declare that the research was conducted in the absence of any commercial or financial relationships that could be construed as a potential conflict of interest.
